# Timing the evolution of antioxidant enzymes in cyanobacteria

**DOI:** 10.1038/s41467-021-24396-y

**Published:** 2021-08-06

**Authors:** Joanne S. Boden, Kurt O. Konhauser, Leslie J. Robbins, Patricia Sánchez-Baracaldo

**Affiliations:** 1 School of Geographical Sciences, University of Bristol, Bristol, United Kingdom; 2 Department of Earth and Atmospheric Sciences, University of Alberta, Edmonton, AB Canada; 3 Department of Earth and Planetary Sciences, Yale University, New Haven, CT USA; 4 Department of Geology, University of Regina, Regina, SK Canada

**Keywords:** Phylogenetics, Geochemistry

## Abstract

The ancestors of cyanobacteria generated Earth’s first biogenic molecular oxygen, but how they dealt with oxidative stress remains unconstrained. Here we investigate when superoxide dismutase enzymes (SODs) capable of removing superoxide free radicals evolved and estimate when Cyanobacteria originated. Our Bayesian molecular clocks, calibrated with microfossils, predict that stem Cyanobacteria arose 3300–3600 million years ago. Shortly afterwards, we find phylogenetic evidence that ancestral cyanobacteria used SODs with copper and zinc cofactors (CuZnSOD) during the Archaean. By the Paleoproterozoic, they became genetically capable of using iron, nickel, and manganese as cofactors (FeSOD, NiSOD, and MnSOD respectively). The evolution of NiSOD is particularly intriguing because it corresponds with cyanobacteria’s invasion of the open ocean. Our analyses of metalloenzymes dealing with reactive oxygen species (ROS) now demonstrate that marine geochemical records alone may not predict patterns of metal usage by phototrophs from freshwater and terrestrial habitats.

## Abstract (web summary)

How early photosynthesizers managed oxidative stress remains relatively unresolved. Analyses of enzymes dealing with reactive oxygen species traces the evolutionary history of superoxide dismutases and finds evidence of CuZnSOD in the ancestor of all cyanobacteria, dating back to the Archaean.

## Introduction

Oxygen is essential for complex life forms as it is used during aerobic respiration to create more energy per mol of substrate than other available electron acceptors^1^. Although today the Earth’s atmosphere contains ~21% oxygen (O_2_), it was at least 10^5^ times lower in the Archaean 4.0–2.5 billion years ago (Gya)^2,3^. Just how and when O_2_ first appeared as a byproduct of biological evolution-oxygenic photosynthesis - remains controversial, with estimates ranging from near the origin of life^4^ to 3.8 billion years ago (Ga)^5^ to immediately preceding the Great Oxidation Event (GOE)^6^, which is estimated to have begun 2.50–2.45 Ga^7,8^. Before this, O_2_ could have been produced by physical processes, such as photodissociation of water and carbon dioxide by UV light^9,10^, but is unlikely to have accumulated at appreciable levels. With the evolution of oxygenic photosynthesis came the potential to produce O_2_ on a much larger scale. Since O_2_ is highly reactive, early Cyanobacteria—the first producers of biogenic O_2_—likely experienced selective pressure, resulting in the evolution of more efficient antioxidants. Such effects have been documented in the photosynthetic machinery, which has been evolving strategies of dealing with reactive oxygen species (ROS) throughout its history^11^. Therefore, it is reasonable to assume that O_2_-generating organisms, such as cyanobacteria, would have co-evolved more efficient mechanisms of managing ROS as water oxidation proteins evolved.

Cyanobacteria remove ROS using carotenoids, α-tocopherol, and antioxidant enzymes, including peroxidases, catalases, superoxide reductases (SORs), and superoxide dismutases (SODs)^12^, and their evolutionary history can be elucidated with phylogenetic methodologies. Although peroxidases and catalases enhance the rate of removal of peroxides (such as H_2_O_2_ and R-O-O-H)^13^, SORs and SODs remove superoxide free radicals (O_2_^.−^)^14^. These O_2_^.−^ are produced as a byproduct of photosynthetic and respiratory electron transport chains^12^ as well as extracellular processes on the cell surface^15^. They can also have beneficial roles in iron acquisition, cell signaling, and growth^15,16^, but if O_2_^.−^ are allowed to accumulate inside the cell, they react with solvent-exposed 4Fe-4S clusters in proteins, including those required for amino acid biosynthesis^17^ and photosynthesis^18^, generating reactants of the Fenton reaction, which can ultimately lead to extensive DNA damage^12^. To balance the beneficial effects of O_2_^.−^ with damage caused by over-exposure, organisms must maintain control over their abundance. Perhaps, it is for this reason that SODs and SORs have been found in all three domains of life—Eukarya, Archaea, and Bacteria^14^.

Phylogenetic methodologies coupled with protein structural analyses have revealed that three different isoforms of SOD enzymes evolved independently of one another to remove unnecessary O_2_^.−^ ^19^. Each has a unique 3D structure, amino acid sequence, and metal cofactor(s); either manganese (MnSOD), nickel (NiSOD), or a combination of copper and zinc (CuZnSOD)^19,20^. A fourth SOD enzyme shares its evolutionary heritage with MnSOD, but utilizes iron as its cofactor (FeSOD)^21^. All of these SODs are found within cyanobacteria^22,23^, but their locations within the cell differ. FeSOD and NiSOD are cytoplasmic^22,24–27^, whereas CuZnSODs from *Synechococcus* and MnSODs from *Anabaena* and *Plectolyngbya* are tethered to membranes^25,27–30^. The choice of which SOD isoform(s) and associated metal cofactor(s) are used by a given species could reflect its evolutionary heritage, function, and environmental history.

The evolutionary record of cyanobacteria spans at least 1.88 Ga (the oldest undisputed colonial cyanobacterial microfossils;^31^), and perhaps as many as or >3.22 Ga (based on stromatolite fabric^32^ and molecular clock analyses of PSII^33^). During this time, cyanobacteria have diversified into a wide range of marine, freshwater, and terrestrial habitats^34,35^. Early phylogenetic studies on the evolution of SODs in cyanobacteria were limited by the availability of genomes but found that NiSODs are encoded in the genomes of planktonic marine strains (such as *Prochlorococcus* spp.), whereas MnSOD and FeSOD are more widespread^22,23,36^. CuZnSODs, while rare, are not restricted to any particular group^22,23^. The 3D structures required for metalloproteins to incorporate copper cofactors have previously been predicted to have arisen after those required to incorporate Fe- and Mn-utilizing proteins in the Proterozoic (~2.4–0.5 Ga), following the GOE^37^. Therefore, it would follow that cyanobacteria used FeSODs and MnSODs before CuZnSODs. Although genes encoding CuZnSODs and FeSODs/MnSODs are widely distributed in bacteria and eukaryotes, NiSODs are restricted to bacteria and may have appeared later in the evolutionary history of life^19^. Previous approaches aimed at determining metal usage in SODs have not considered the habitats where strains live or evolved. This is problematic because metal availability differs across oceanic and terrestrial environments^38,39^.

Here, we analyze when cyanobacteria acquired genes encoding NiSOD (*sodN*), CuZnSOD (*sodC*), FeSOD (*sodB*), and MnSOD (*sodA*) in the context of their habitat and metal cofactor availability through geological time. To do this, we construct phylogenies of all SODs encoded in the genomes of 15,899 bacteria. We also implement a phylogenomic approach and multiple molecular clock analyses to estimate when the crown group of cyanobacteria diverged from their closest non-photosynthetic relatives, the Vampirovibrionia^40,41^. By studying the timing of the divergence of cyanobacteria from their closest relatives, as well as their acquisition of SODs, we establish points in the Proterozoic and Archaean when these SODs were present in cyanobacteria.

## Results

### Bacterial SOD diversity

**Fig. 1:**
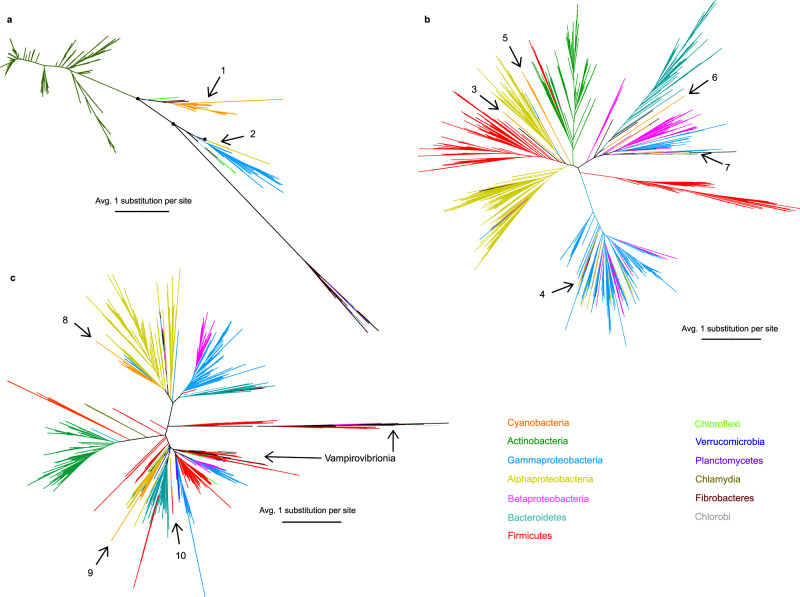
Evolutionary relationships of superoxide dismutases in bacteria. **a** NiSOD **b** CuZnSOD **c** Fe- and Mn-utilizing SODs. **a** and **b** are representative of three independent replicates (Supplementary Figs. 11–12), whereas **c** is the most likely of three potential topologies (Supplementary Fig. 1). All trees were constructed with ML methodology implemented in IQ-TREE v1.6.1^46^. Branches are colored to represent proteins found in 1 of 13 bacterial phyla: Cyanobacteria (orange), actinobacteria (green), gammaproteobacteria (blue), alphaproteobacteria (yellow), betaproteobacteria (pink), bacteroidetes (cyan), firmicutes (red), chloroflexi (light green), verrucomicrrobia (dark blue), planctomycetes (purple), chlamydia (olive), fibrobacteres (dark green), and chlorobi (gray). Branch lengths represent the number of amino-acid substitutions per site with scale bars representing an average of one substitution per site. Numbered arrows indicate cyanobacterial proteins. Interesting UFBoot values ≥ 95 in all NiSOD replicate trees are indicated with black circles.

To elucidate which transition metals cyanobacteria first used to protect themselves against the oxidative stress caused by O_2_^.−^, the evolutionary history of SOD genes was modeled and mapped onto an updated time-calibrated phylogeny. In order to do this, we began by screening 15,899 bacterial genomes for genes encoding NiSOD, CuZnSOD, and MnSOD. BLASTP was unable to distinguish between genes encoding FeSOD and MnSOD, so they are considered as one hereafter. Results reveal that the *sodN* gene encoding NiSOD is less common in bacteria than *sodC* encoding CuZnSOD. The *sodN* gene was found in 1,464 different species, including gammaproteobacteria, alphaproteobacteria, planctomycetes, bacteroidetes, actinobacteria, cyanobacteria, verrucomicrobia, and chloroflexi (Fig. 1a). By contrast, *sodC* was absent from the latter two phyla, but present in more genomes (5,723) of beta-, gamma- and alpha–proteobacteria as well as planctomycetes, bacteroidetes, actinobacteria, cyanobacteria, and firmicutes (Fig. 1b). Together, *sodA* and *sodB* are more widespread than *sodC* and *sodN* combined, being present in 13,748 bacterial genomes across all ten phyla mentioned previously, as well as fibrobacteres, chlorobi, and vampirovibrionia (Fig. 1c and Supplementary Fig. 1).

### Cyanobacterial SOD diversity

**Fig. 2:**
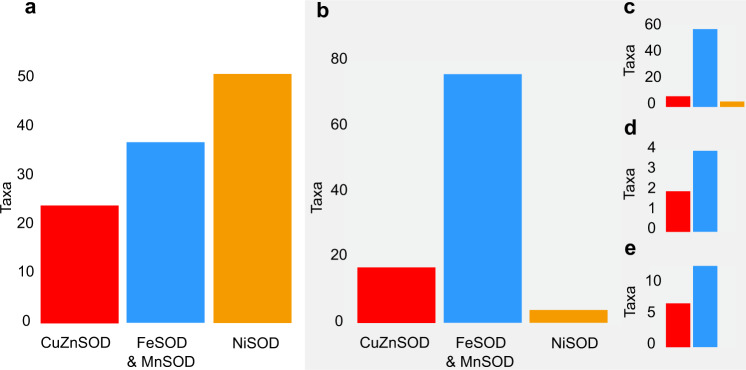
Habitat distribution of SOD genes. Panels represent the distribution of CuZnSOD (red), NiSOD (orange), and Fe- and Mn-utilizing SODs (blue) in cyanobacteria from **a** marine and **b** non-marine habitats. Non-marine habitats include **c** freshwater, **d** geothermal springs, and **e** terrestrial habitats.

**Fig. 3:**
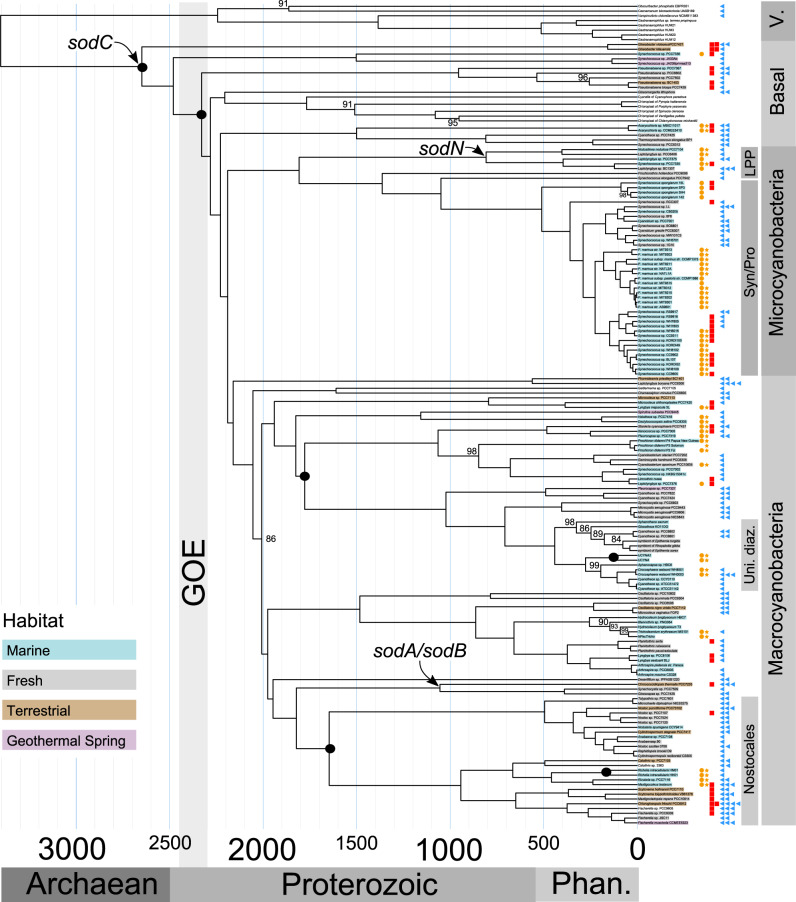
Time-calibrated cyanobacterial tree of life and superoxide dismutases. Genes encoding NiSOD (orange circles), NiSOD maturation protease (orange stars), CuZnSOD (red squares), and SODs with Mn- or Fe- cofactors (blue triangles) are highlighted next to the strain names. The earliest node predicted to have *sodN, sodC* and *sodA* or *sodB* are annotated with labels (see Table 2 for posterior age probabilities and Supplementary Fig. 10 for the age distribution of these events). The phylogenetic tree was estimated from SSU and LSU ribosomal RNA and 136 core cyanobacterial proteins from 167 different taxa using the maximum likelihood methodology implemented in IQ-TREE v1.6.1^46^. Node labels represent ultrafast bootstrap approximations <100^98^. Ages were estimated using a Bayesian relaxed molecular clock with Uncorrelated Gamma Multipliers^49^ for ribosomal RNA. Black circles represent calibration points (Table 1). The first divergence of cyanobacteria was constrained to occur between 2.7 Ga and 2.32 Ga^110,111^. The color behind each strain name indicates whether it is from a marine habitat (light blue), freshwater habitat (gray), terrestrial habitat (light brown), or geothermal spring (light pink). *GOE* Great Oxidation Event, *Uni. diaz.* unicellular diazotrophs, *Phan.* Phanerozoic eon, *V.* Vampirovibrionia.

A more specific search among cyanobacteria revealed that most strains with *sodN* (51 of 55 strains) live in saltwater habitats (Fig. 2). They include representatives from all major clades of marine taxa (Fig. 3). Ten lack a gene (named *sodX*) encoding NiSOD’s maturation protease (Supplementary Data 1). This maturation protease activates NiSOD^42^ by cleaving the preprotein^43^, so these strains may not be able to use NiSOD to remove O_2_^.−^. Six of them contain genes encoding other SOD isoforms, while the remaining four picocyanobacteria (i.e., *Prochlorococcus* and *Synechococcus* species) do not (Fig. 3). The genome completeness of free-living strains—measured with BUSCO v3.0.2 using lineage data from cyanobacteria^44^—is estimated at 92–98% so it is possible that the maturation protease gene is present but has not been sequenced (Supplementary Table 1).

Many cyanobacterial strains contain multiple SOD isoforms. These include 32 strains with *sodN*, every strain with *sodC*, and 45 of 115 with *sodA* or *sodB*. The remaining 23 strains which only use NiSOD (including three without *sodX*) are all marine with small genomes. They include picocyanobacteria and endosymbionts living in larger marine tunicates, algae, and sponges (e.g., *Prochloron* spp., UCYNA, and two strains of *Synechococcus spongiarum*). The 70 cyanobacteria which only use FeSOD and MnSOD live in a variety of marine, terrestrial, and freshwater habitats (Fig. 3). Only 3% of cyanobacteria (five of 149 strains discounting plastids) have genes encoding every SOD isoform as well as the NiSOD maturation protease (Fig. 3). Paralogues of *sodC* were found in three genomes and paralogues of *sodA* and/or *sodB* were found in at least 13 genomes (Supplementary Data 1).

The resources needed to make each SOD isoform vary. NiSOD is composed of a mode of 157 amino acids (range 145–166), whereas CuZnSOD is composed of 177 (range 103–236) and FeSOD/MnSOD of 200 (range 197–280) (Supplementary Fig. 2).

### Transfer of SODs across phyla

To investigate whether cyanobacteria obtained their SOD genes from other bacterial phyla, maximum likelihood (ML) phylogenies were constructed using the NiSODs, CuZnSODs, and combined Mn- and Fe-SODs from bacteria. If, in each case, all cyanobacterial proteins form a monophyletic group (i.e., they share a recent common ancestor), this would indicate a single origin. Surprisingly, these phylogenetic analyses revealed that cyanobacterial NiSODs, CuZnSODs, MnSODs, and FeSODs are polyphyletic (Fig. 1 and Supplementary Fig. 1), suggesting independent origins perhaps due to several lateral gene transfer events or other modes of reticulated evolution. Furthermore, the SODs with Mn or Fe cofactors found in vampirovibrionia are not related to those in their sister phyla, cyanobacteria^40^ (Fig. 1c, Supplementary Table 2 and Supplementary Fig. 1).

Molecular phylogenies identified several horizontal gene transfers (HGTs) of each SOD isoform between cyanobacteria and other bacterial phyla: Two of *sodN* (NiSODs), five of *sodC*, and three to eight of Mn- and Fe-utilizing SODs (Fig. 1 and Supplementary Fig. 1). A variety of different phyla are involved in these HGTs. For example, most cyanobacterial NiSODs have diversified from a protein resembling that of benthic marine Deltaproteobacteria (namely *Geopsychrobacter electrodiphilus*, UFBoot 88 and *Plesiocystis pacifica*, UFBoot 89, Supplementary Fig. 3), whereas other cyanobacterial NiSODs (present in *Synechococcus spongiarum*) diversified from a protein resembling those of alphaproteobacteria (UFBoot 81, Supplementary Fig. 4).

Genes encoding CuZnSOD may have been present in the shared common ancestor of all extant cyanobacteria. This ancestor gave rise to basal lineages before diverging into macrocyanobacteria and microcyanobacteria (Fig. 3). Although CuZnSODs are rare in macro- and micro–cyanobacteria, they are present in most free-living basal lineages (Fig. 3). Two in particular (*Pseudanabaena* spp. and *Gloeobacter* spp.) share *sodC* genes which are monophyletic (PP 1) and closely related in the same way as the species are to one another (Supplementary Fig. 5). This suggests they have been vertically inherited from the common ancestor of all extant cyanobacteria (otherwise known as the cyanobacteria crown group).

As cyanobacteria diversified to occupy new ecological niches and habitats^34^, the *sodC* genes which initially allowed crown cyanobacteria to use copper and zinc to protect against oxidative stress were likely lost. Later, HGTs may have occurred between non-cyanobacterial phyla and picocyanobacteria, diazotrophs, sponge symbionts, *Acaryochloris* spp. and *Microcoleus chthonoplastes* (Fig. 1), resulting in the distribution found today.

### Bayesian relaxed molecular clock analyses

Divergence times were estimated in Phylobayes 4.1^45^ using SSU and LSU ribosomal RNA from 164 cyanobacteria and eight vampirovibrionia. The molecular clock’s topology was constrained using an ML tree constructed in IQ-TREE v1.6.1^46^ from these same ribosomal RNAs plus 136 core cyanobacterial proteins with similar evolutionary trajectories^47^. They have a range of functions in metabolism, cellular processes, and information handling^34,35,48^. All molecular clock analyses implemented six calibration points (Table 1) as previously described^48^. Divergence times were estimated using uncorrelated gamma multipliers^49^. All analyses, regardless of calibration points and models, indicate that cyanobacteria diverged from vampirovibrionia between 3.3 and 3.6 Ga in the Archaeaneon (Table 2). Estimates that include 95% credibility intervals allow for a range between 2.8 and 4.3 Ga, suggesting that cyanobacteria diverged from their sister phyla at at least 300 million years before the GOE (Table 2).

**Table 1:** Calibration points used for molecular clock analyses.

Calibration	Minimum age/mya	References	Maximum age /mya	References	Diversification between…
1st cyanobacterial diversification	2320	^110^	2700	^111^	*Gloeobacter violaceus* PCC 7421
	2320	^110^	3000	^56^	*Acaryochloris* sp. MBIC 11017
Filamentous cyanobacteria	1900	^103^	n/a	n/a	*Pseudanabaena biceps* PCC 7429
					*Leptolyngbya* sp. PCC 7376
Akinete-forming cyanobacteria	1600	^105^	1888	^102,104^	*Calothrix* sp. PCC 7103
					*Nostoc azollae* 0708
Apical cells of cyanobacteria	1700	^106^	1888	^102,104^	*Pleurocapsa* sp. PCC 7327
					*Pleurocapsa* sp. PCC 7319
Endosymbionts of *Hemiaulus* spp.	110	^108^	n/a	n/a	*Richelia intracellularis* HH01
					*Richelia intracellularis* HM01
Endosymbionts of *B. begelowii*	91	^109^	n/a	n/a	*UCYNA2*
					*UCYNA*

Note: Genus and species names are italicized.

**Table 2:** Statistics summarizing divergence times predicted by molecular clock analyses.

	First divergence of cyanobacteria*	
	3–2.32 Ga	2.7–2.32 Ga
Divergence of cyanobacteria from vampirovibrionia	3544 (4235–3001)	3374 (4058–2807)
NiSOD	912 (1982–281)	806 (1898–226)
CuZnSOD	2926 (3043–2692)	2649 (2720–2490)
MnSOD/FeSOD	1140 (1941–207)	1045 (1893–171)

Mean divergence times are presented in millions of years alongside 95% confidence intervals in brackets.

* describes the calibration strategy used to constrain the first radiation of cyanobacteria, where Gloeobacter spp. diversify from other cyanobacteria.

## Discussion

The advent of oxygenic photosynthesis had profound impacts on Earth’s climate, chemistry, and biota. It destroyed a methane greenhouse which kept the world warm^50^, enhanced the supply of sulfate and redox-active metals to the oceans^51,52^, marginalized the growth of anoxygenic phototrophs^53^, and facilitated the chemical oxidation of dissolved Fe(II) in seawater to form banded iron formations (BIF)^54^. Organisms would have responded by choosing more bioavailable metals where possible and evolving more efficient enzymes, such as SODs, to deal with the expansion of O_2_ into new habitats^34^.

Age estimates for the emergence of oxygenic photosynthesis are therefore fundamental to reconciling the biochemistry and use of trace metals. Previous estimates place the divergence of cyanobacteria and their closest relatives, vampirovibrionia^40,42^, in the neoarchaean between 2.5 and 2.6 Ga^55^, but our molecular clock analyses point to an earlier origin where the cyanobacterial lineage emerges in the Paleoarchaean between 3.3 and 3.6 Ga based on mean predicted ages (95% confidence intervals range from 2.8 to 4.2 Ga) (Table 2). The difference between these estimates is owing to the choice of molecular clock model, variations in taxa selection, and alternative molecular markers of age divergence. Calibration points are also fundamental for predicting macroevolutionary patterns over long geological timescales. Our clocks are calibrated with cyanobacterial microfossils, whereas Shih, et al.^55^ are calibrated with non-cyanobacterial fossils. Our finding an earlier origin of cyanobacteria are thus consistent with geochemical studies showing that oxygen was accumulating in shallow coastal habitats ca. 3.0–3.2 Ga^32,56,57^ and molecular clock analyses of the PSII gene family which predict that photosystems capable of splitting water arose early in the history of life on Earth^33,58^. We also note that our estimate of 3.3–3.6 Ga is consistent with a recent survey of oxygen utilizing and producing enzymes (e.g., oxygenases) which predicts the rapid proliferation of these enzymes 3.1 Ga^59^; although differences arise in the dating of FeSOD and MnSOD between this study and that of ref. ^59^ (see below for discussion).

The action of photosystems (like some other molecules, such as transmembrane NADPH oxidases and flavins) produces O_2_^.−^ ^14^ and when production is particularly high (owing to bright light for example), SODs protect the photosynthetic repair mechanisms from damage^25,60,61^. Key parts of this photosystem repair mechanism (namely membrane-bound FtsH proteases which degrade damaged photosystem components) evolved with the advent of oxygenic photosynthesis in the Archaean^62^. Our phylogenetic analyses suggest for the first time that CuZnSOD, an antioxidant enzyme capable of protecting photosynthetic repair mechanisms from oxidative damage^25^, was also present in cyanobacteria in the Archaean, at least 2.9–2.6 Ga (Table 2). This fits with alternative phylogenetic analyses predicting that *sodC* was present in the last universal common ancestor of life on Earth^63^.

Our conclusion is based on the close relationship of CuZnSODs from two basal lineages: *Pseudanabaena* spp. and *Gloeobacter* spp. (Supplementary Fig. 5). The most recent common ancestor (MRCA) of these two genera appeared ~3.4 Ga (confidence intervals span 4.2–2.8 Ga), and diversified ~2.6 Ga (confidence intervals span 3.0 to 2.5 Ga), giving rise to all extant cyanobacteria (Fig. 3). If the ancestor of crown cyanobacteria had *sodC*, it could have been inherited by its descendants, resulting in a unique phylogenetic signal whereby the CuZnSODs of *Gloeobacter* spp. and *Pseudanabaena* spp. are sisters. Because this phylogenetic pattern exists (Supplementary Fig. 5), it is likely that Archaean cyanobacteria used CuZnSOD. An alternative scenario could result in the same pattern if *sodC* was transferred laterally from the MRCA of *Pseudanabaena* spp. to the MRCA of *Gloeobacter* spp. This would put an upper age constraint on the origin of CuZnSOD ~ 0.95 Ga (confidence intervals span 2.2 to 0.3 Ga) (Fig. 3).

Although *sodC* has been transferred between bacterial phyla on multiple occasions in the past (Fig. 1b), our phylogenetic analyses provide less evidence of HGT between cyanobacteria of terrestrial and freshwater origin (see Supplementary discussion). As the MRCAs of *Pseudanabaena* and *Gloeobacter* spp. were not marine (Supplementary Fig. 6), HGT accompanied by a more recent origin of CuZnSOD ~ 0.95 Ga seems unlikely, if not impossible.

**Fig. 4:**
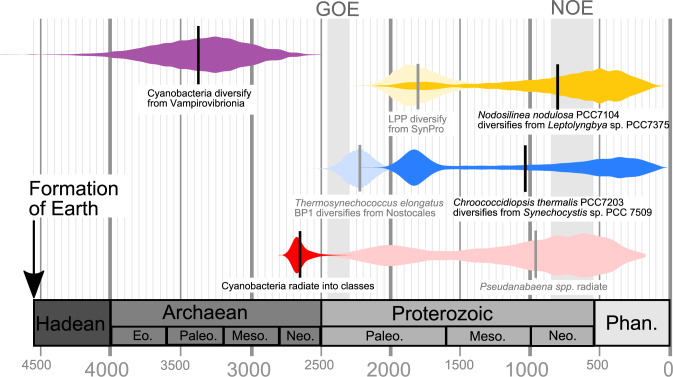
Emergence of cyanobacteria (purple) and their CuZnSODs (red), NiSODs (orange), and Mn- and/or Fe-utilizing SODs (blue) inferred through geological time. Coloured bands represent the distribution of age posterior estimates (Table 2) for key nodes in a Bayesian molecular clock parameterized with uncorrelated Gamma Multipliers^49^ and calibrated with the first radiation of cyanobacteria between 2.32 and 2.7 Ga. Vertical black lines represent the mean in each instance. Dark-colored bands represent the earliest cyanobacteria predicted to have genes encoding the relevant SOD isoform based on a hypothesis of vertical inheritance, whereas paler nodes require horizontal gene transfer or other patterns of reticulated evolution as described in the text. *Phan* Phanerozoic eon; *Meso* Mesoproterozoic era; *Neo* Neoproterozoic era; *GOE* Great Oxygenation Event, *NOE* Neoproterozoic Oxygenation Event.

Further insight can be gained by timing the origin of Cu-based metalloenzymes in general. Characteristic protein-folds required to bind Cu are predicted to have evolved during, or following, the GOE^37^. However, geological evidence suggests that metabolisms which rely on copper metalloenzymes (e.g., nitrification), were present in the Archaean, 2.7 Ga^64^. If proteins could incorporate Cu cofactors in the Archaean, then CuZnSODs could have been present at the root of cyanobacteria as a mechanism to deal with O_2_ generated by photosynthesis before it accumulated in the global atmosphere (at the GOE). This would have been particularly important for Mesoarchaean and Neoarchaean individuals living inside benthic mats^65,66^, where there was a smaller diffusion gradient to pull O_2_ out of the cells. As a result, higher concentrations of O_2_ accumulated, raising the potential for superoxide radical generation, particularly in the periplasm^67^. In the alternative (if unlikely) scenario where basal cyanobacteria acquire *sodC* via HGT ~ 0.95 Gya, Archaean cyanobacteria might not have used SOD to remove O_2_^.−^ because we do not find any phylogenetic evidence for NiSOD, MnSOD, or FeSOD having been inherited from an ancestor present before the GOE (Fig. 4). Two scenarios that could reconcile an earlier origin of NiSOD and Fe- or Mn-utilizing SODs with our evolutionary trees (Supplementary Figs. 7–8) are presented in the Supplementary Discussion. All rely on more extensive HGT, so age distributions are presented in Fig. 4, but in paler colors, because most prokaryotic proteins are transferred via vertical inheritance^68–70^.

Why are there only a few cyanobacterial strains with the gene encoding CuZnSOD today? Perhaps it is related to physiological considerations resulting in the replacement of CuZnSOD for MnSODs. The thylakoid membranes of *Nostoc* sp. PCC7120 lack CuZnSOD (Fig. 3) and instead contain MnSOD^30^. When exposed to intense sunlight these MnSODs protect the cell from photoinhibition^60^ in a similar manner to CuZnSOD^25^. Furthermore, the gene encoding MnSOD can be post-translationally processed to produce three proteins of different sizes which vary in concentration between heterocysts and vegetative cells^29^. Under nitrogen-supplemented conditions, the smallest cytosolic 24 kDa protein predominates the slightly larger 27 kDa protein, but under nitrogen-limiting conditions, both proteins are present in near equal proportions^29^. No such flexibility has been documented in CuZnSODs. Therefore, the ability to modify the size and localization of MnSOD proteins in response to changes in the environment could explain why the *sodA* gene encoding MnSOD is present in all major clades of nitrogen-fixing cyanobacteria (defined by^71^, including all Nostocales, one *Trichodesmium* spp., seven unicellular diazotrophs, *Chroococcidiopsis thermalis,* and *Leptolyngbya* sp. PCC7375, Fig. 3) and many phyla of non-photosynthetic bacteria with diazotrophic representatives (e.g., Firmicutes, Actinobacteria and Proteobacteria^72^, Fig. 1c and Supplementary Fig. 1). Phylogenetic evidence for the utilization of SODs that incorporate Fe and Mn cofactors appears relatively recently, in the middle of the Proterozoic (Table 2, Fig. 4). Intriguingly, modern cyanobacteria might be less sensitive to oxidative stress caused by O_2_^.−^ because their proteins with 4Fe-4S clusters have evolved to protect themselves by positioning their Fe atoms in less-solvent-exposed parts of the molecule. It may be with this method that picocyanobacteria are able to thrive with only a cytoplasmic NiSOD (Fig. 3).

The relatively late appearance of FeSOD and MnSOD in cyanobacteria is surprising as previous studies have postulated an Archaean origin in bacteria^19,59,73,74^. What caused this delay? Earth system models suggest that photoferrotrophs outcompeted cyanobacteria for upwelling nutrients in aquatic habitats prior to the GOE^53,75^. Perhaps, they also limited the soluble Fe^2+^ available for cyanobacteria, thus facilitating a selective advantage to lineages that used alternative metals for relieving oxidative stress. As the atmosphere became more oxygenated, photoferrotrophs were marginalized to shrinking pools of Fe^2+^. Our analyses suggest that the Neoproterozoic oxygenation increased cyanobacteria’s requirement for ROS defense mechanisms so much that lineages began using FeSOD or MnSOD regardless of the waning global concentrations of Fe and Mn (Fig. 4). Further study, however, will be needed to assess how effectively FeSODs, MnSODs, and CuZnSODs protect against oxidative stress under different ambient concentrations of oxygen and transition metals.

Phylogenetic evidence also indicates that cyanobacteria started using cytosolic NiSOD isoforms in the MRCA of *Leptolyngbya* sp. PCC7375 and *Nodosilinea nodulosa* PCC7104, which diversified in marine Neoproterozoic (Table 2, Fig. 4) habitats (Supplementary Fig. 6). Earlier, during the Paleoproterozoic and Mesoproterozoic, phylogenomic evidence suggests that cyanobacteria were living in terrestrial, coastal brackish, and marine benthic environments^34,76^. Therefore, NiSOD usage also corresponds with the first appearance of marine planktonic unicellular nitrogen-fixing cyanobacteria and non-nitrogen-fixing picocyanobacteria (including many *Synechococcus* spp. and all *Prochlorococcus* spp.) in the open ocean at the end of the Precambrian^35,48,77,78^. Although only speculative at this time, it is possible that the acquisition of NiSOD and its associated maturation protease assisted the invasion of cyanobacteria into pelagic marine habitats. Pelagic planktonic cyanobacteria are typically limited by P, Fe, and N (in cases of non-nitrogen fixers) owing to their distance from sources of riverine discharge^79^. As NiSOD is composed of fewer amino acids than other SOD isoforms (Supplementary Fig. 2) and does not require Fe, its utilization by benthic marine cyanobacteria in the Neoproterozoic may have imparted an evolutionary advantage and increased their resilience to nutrient limitation in the open ocean.

By studying only extant taxa, it is inherently difficult to estimate whether extinct lineages of cyanobacteria used NiSOD, MnSOD, FeSOD, or CuZnSOD prior to the estimations provided in Fig. 3, Fig. 4, and Table 2. It is reassuring, however, that our estimates of all SOD isoforms predate the Cretaceous-Paleogene mass extinction of non-avian dinosaurs from terrestrial and aquatic environments 66 Mya^80^, the Permian-Triassic mass extinction of 56% of all marine animal genera 252 Mya^81^, and the Snowball Earth glaciations 720–635 Mya, which subjected life to extreme climate fluctuations^82^. Therefore, our estimations of timing are robust across several past extinction events. Any future discovery of novel lineages of basal cyanobacteria, however, could alter our estimated order of SOD appearance.

It has been proposed that trace metal inventories of ancient marine sediments should reflect trace metal availability and correspond to the emergence of novel metabolisms and metalloenzymes^37^. In light of this suggestion, the past two decades have seen a number of papers purporting the use of ancient marine chemical sediments (i.e., BIF, shales, pyrite) to track seawater composition through time (see ref. ^39^ for a review). With regards to the metal cofactors contained in SOD, their temporal trajectories have been reconstructed based on both thermodynamic considerations (e.g.^83^) and sedimentary records (e.g.,^84,85^). However, there is a poor record of trace metal inventories outside of marine habitats, so it can be more difficult to establish links between metal availability and diversification of cyanobacteria living in terrestrial and freshwater habitats.

Our ancestral state reconstructions predict that a non-marine cyanobacterium could have acquired CuZnSOD (Supplementary Fig. 6) in the Archaean (Table 2). In the Archaean to Proterozoic ocean, Cu and Zn were supposed to have been present at exceedingly low abundances given the low solubility of sulfide phases and the belief that they would be sequestered into euxinic sediments after the GOE. However, BIF and shale records indicate near static reservoirs for Zn^84,85^ and Cu^86^ in the water column. This may hint at either a strong biological control on water column cycling being established very early on or the possible complexation of these trace metals by organic ligands. Although the possible release of Cu through the oxidative weathering of sulfides in the lead up to, and following, the GOE^86^, may explain the emergence of CuZnSOD in the Neoarchaean, at this point there is a dearth of geochemical evidence to support this hypothesis, at least from a global first-order perspective. Despite this, existing chemical sediment compilations cannot preclude the possibility of local or transient increases in Cu, creating oases of bioavailability that stimulated the emergence of CuZnSODs.

**Fig. 5:**
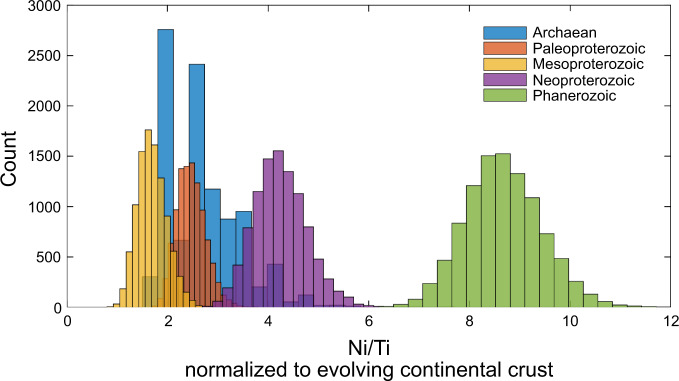
Bootstrap resampled mean values from time-binned shale samples. Each group represents a time-binned subset of the shale database representing the Archaean (blue), Paleoproterozoic (orange), Mesoproterozoic (yellow), Neoproterozoic (purple), or Phanerozoic (green) with mean values of molar Ni/Ti, normalized to evolving crust^116^, being bootstrap resampled (*n* = 10,000 for each bin).

Past reconstructions of Ni in seawater based on BIF and syn-sedimentary to early diagenetic pyrite^87–89^ show a dramatic, and unidirectional, decline that immediately preceded the Great Oxidation Event. This drop has been proposed to reflect a decline in the flux of nickel to the oceans due to waning komatiite weathering and would have marginalized methanogenic microbial communities from the water column to the sediment pile, corresponding to seawater concentrations decreasing from as high as 400 nM down to effectively near modern levels of Ni (~ 9 nM)^87,88^. In turn, this would have created environmentally favorable conditions for a rise in oxygenic photosynthesis by cyanobacteria. This Neoarchaean to Paleoproterozoic decline in Ni is also reflected in a compilation of shale data, normalized to evolving upper continental crust (Fig. 5). Interestingly, however, there appears to be a slight uptick in the flux of Ni from continental weathering to the oceans in the terminal Neoproterozoic to Phanerozoic as evidenced by elevated Ni/Ti ratios (Fig. 5) that occurs shortly after the appearance of NiSOD in the Tonian period (Table 2), which is not captured in the BIF record^87,88^. This discrepancy in sedimentary records is likely due to the declining concentration of Fe in seawater through time as Earth’s oceans moved from a dominantly anoxic and ferruginous state in the Archaean to the modern oxic oceans we have today; a trend perhaps best reflected in the disappearance of large superior-type BIF following the Paleoproterozoic (e.g.,^90^).

Elevated Ni/Ti ratios during the Neoproterozoic presumably reflects a relatively greater Ni flux to the oceans from either increased oxidative weathering, similar to other sulfide bound metals such as molybdenum or vanadium at this time^52,91^, or through the weathering of freshly emplaced large igneous provinces as has been proposed for phosphorous^92^. At present, the exact mechanisms and timing of higher Ni concentrations in Neoproterozoic shales require further investigation as it lags behind the observed increases in molybdenum, vanadium, and phosphorus^52,91–93^.

Given the histories of these trace metals, one may ask whether it is availability driving the assimilation of Cu, Zn, Fe, Mn, and Ni into SOD metalloenzymes, or an inherent competition with other extant lineages at any given time. For instance, metalloenzyme fold superfamilies that incorporate Zn proliferate late and have been proposed to contribute to the delay in eukaryotic evolution^37^. Alternatively, this has been proposed to reflect an intrinsic biological property of eukaryotic evolution rather than a sudden shift in Zn availability^84^. In the latter view, the early incorporation of Cu and Zn into SODs would reflect a lack of competition from the yet unevolved eukaryotes in the Archaean. Then in the late Mesoproterozoic, as eukaryotes began to become more abundant and dominate marine and terrestrial environments, they may have outcompeted cyanobacteria for Cu and Zn, providing an impetus for the emergence of other membrane-bound SODs, such as MnSOD—effectively providing a strategy for alleviating limitation imparted by competition with emergent eukaryotes. Although eukaryotes also utilize Mn, protein structures required to bind Mn have not been preferentially retained in their genomes^94^ and cyanobacteria, having evolved earlier than photosynthetic eukaryotes^95^, had plenty of time to develop an efficient Mn uptake system.

Later, in Neoproterozoic marine habitats, cyanobacteria began supplementing their ROS defense mechanisms with a new and smaller SOD isoform (NiSOD) that requires less phosphorus and nitrogen to manufacture. This timing of *sodN* incorporation into the cyanobacterial genomic repertoire coincides with Earth’s second planetary oxygenation^3^ and the emergence of planktonic marine cyanobacteria^35^. Therefore, we suggest that cyanobacteria began assimilating nickel into SODs to invade Neoproterozoic planktonic communities as Neoproterozoic oxygen levels rose.

## Methods

### Acquisition of SOD sequences

Bacterial SOD diversity was assessed by downloading 15,899 bacterial genomes from the NCBI RefSeq database (https://www.ncbi.nlm.nih.gov/refseq/) in 2018. They were searched for genes encoding NiSOD, CuZnSOD, FeSOD, and MnSOD using the basic local alignment search tool (BLAST) for proteins (https://blast.ncbi.nlm.nih.gov/Blast.cgi) with a word size of six, gap opening penalty of 11, gap extension penalty of 1, and *e* value cut off of <1 × 10^−5^. Query sequences are detailed in Supplementary Table 3. They were aligned to relevant subject sequences using the BLOSUM62 substitution matrix. Each genome was treated as a separate subject (functionally equivalent to a unique database) and if multiple homologs of a single SOD isoform were found in a given genome, only one with the highest bitscore was retained, except for the cyanobacteria and vampirovibrionia where all hits were included (Supplementary Data 2). The resulting hit’s functions were verified by choosing a representative subsample of hits with distant evolutionary relationships and noting functional domains predicted by the NCBI conserved domain search (https://www.ncbi.nlm.nih.gov/Structure/cdd/wrpsb.cgi) (Supplementary Data 3 and Supplementary Fig. 9). If possible, query sequences with experimentally defined functions were utilized.

Cyanobacterial SOD diversity was assessed by sampling 153 additional genomes (strains are listed in Supplementary Data 1 and their habitat in Supplementary Table 4), including representatives from all major clades of salt-tolerant and freshwater cyanobacteria^35^. These were supplemented with five eukaryotic plastid genomes^95^ and eight vampirovibrionial genomes representing all four orders (Gastranaerophilales, Vampirovibrionales, Caenarcaniphilales, and Obscuribacterales)^40^.

### SOD gene phylogenies

Amino-acid sequences for all SOD isoforms (including paralogs found in cyanobacteria) were aligned using MAFFT^96^ and gaps present in ≥80% of sequences removed. The evolutionary history of each isoform was estimated by constructing nine ML phylogenies with IQ-TREE version 1.6.1^46^. Substitution models were identified by ModelFinder^97^ and node supports measured using ultrafast bootstrap two approximations^98^.

### Species phylogeny of cyanobacteria

An evolutionary tree of cyanobacteria was generated from a data set including SSU rRNA (1687 nucleotides), LSU rRNA (3387 nucleotides), and 136 core proteins (52,227 aa). Each protein is encoded by an orthologous gene, which is conserved among cyanobacteria and involved in a key cellular function, such as information processing, metabolism, or cellular processes. For a full description of these genes, see previous papers^34,35,77^. Each protein and ribosomal RNA were aligned using MAFFT^96^ and concatenated using an alignment viewer (http://sdsssdfd.altervista.org/arklumpus/AlignmentViewer/AlignmentViewer.html). Gaps were removed if present at the same position in ≥80% sequences and ML methodology was implemented in IQ-TREE v1.6.1^46^ to estimate the cyanobacterial phylogeny. Each protein and rRNA was characterized under a unique partition with -SP to account for heterotachy and appropriate substitution models identified by ModelFinder^97^. Ultrafast bootstrap values were calculated to measure support for branching relationships by resampling sites within partitions 1000 times^98^.

### Divergence time estimation

Bayesian relaxed molecular clocks were implemented using the topology described above. This topology was fixed, and ages estimated based on predicted mutation rates for ribosomal RNA (SSU and LSU, 5074 aligned positions). Substitutions were modeled using a flexible general time-reversible model inferred from the alignment and a Dirichlet process prior^99^ (CAT-GTR) to account for different rates of evolution between distant sites of the molecule. Divergence times were estimated in phylobayes 4.1^45^ using uncorrelated gamma multipliers^,49^, a birth-death prior on divergence times, and root prior chosen from a Gamma probability distribution with mean 3060 and standard deviation 404. As a result, 97% of the prior distribution specified that the ancestor of all vampirovibrionia and cyanobacteria originated after the end of the late heavy bombardment 3.9 Ga^100,101^.

We also implemented microfossil calibrations as follows; filamentous cyanobacteria >1.9 Ga^102,103^, akinete-forming cyanobacteria 1.6–1.888 Ga^102,104,105^, and apical cells of cyanobacteria 1.7–1.888 Ga^102,104,106^. These fossil constraints were supplemented with evidence dating the appearance of eukaryotic hosts of endosymbiotic cyanobacteria. For example, *Richelia* species diversify before their diatom host, named *Hemiaulus*^107^, appeared 110 Mya^108^, and UCYNA species diversify before their prymnesiophyte host, named *Braarudosphaera bigelowii*, appeared 91 Mya^109^. Geochemical evidence was used to constrain cyanobacteria to first diversify before the Great Oxygenation Event of 2.32 Ga^110^, but after either; (a) 3 Ga when molybdenum oxides document the first “whiffs” of atmospheric oxygen^56^; or (b) 2.7 Ga based on the earliest fossilized evidence of cyanobacterial stromatolites^111^. Soft bounds were applied throughout to allow 5% of the prior probability density to fall outside of the specified minimum and maximum ages.

Models were considered complete when four replicate independent chains converged. This was tested by estimating effective sample sizes and relative differences using Tracecomp (in Phylobayes with *effsize* > 50, *reldiff* < 0.3). Chronograms and divergence times were calculated from a single representative chain using Readdiv (in Phylobayes) with the same burn-in (25% of mean chain length) and sampling frequency (1 in every 10 points) as Tracecomp.

### Chronology of SOD isoforms

The order in which SOD isoforms appeared in cyanobacteria, was estimated using a method described as “topological comparison”^112^. First, a Bayesian protein phylogeny was created for each SOD isoform using only cyanobacterial sequences (Supplementary Figs. 5 and 7). Bayesian phylogenetic reconstructions of MnSODs and FeSODs had not converged after 2 weeks, so the sequences were separated into seven groups based on their position in the ML bacterial phylogenies (Supplementary Fig. 1) to conduct alignments and phylogenetic reconstructions on more similar sequences (Supplementary Fig. 8). The resulting protein phylogenies were then compared with the species phylogeny of cyanobacteria (Fig. 3) to identify monophyletic groups whose NiSOD, CuZnSOD, or MnSOD/FeSOD had evolved as expected by vertical inheritance (without HGT). The last common ancestor of each of these clades was assumed to have been capable of using the corresponding SOD.

### Ancestral state reconstruction of habitat preference

To find out which habitats ancestral cyanobacteria lived in when SOD isoforms appeared, Bayesian stochastic character mapping^113^ was implemented with SIMMAP v1.5^114^ in the phytools package^115^ of R using our time-calibrated trees. Prior distribution on the root node of the tree was estimated based on the data, 1000 simulations were performed and the all rates different model was utilized to allow transition rates between marine and non-marine habitats to vary based on the data (as implemented in^95^). Character states were coded as either “marine” or “non-marine” (Supplementary Data 1).

### Compilation of Ni data

From a database of >4000 literature shale analyses spanning the Archaean to modern, 1584 had both Ni and Ti data available and are used here to reconstruct the trajectory of Ni in seawater (Supplementary Data 4). Molar Ni/Ti ratios were normalized to evolving continental crust^116^ then time-binned based on age (Archaean, Paleoproterozoic, Mesoproterozoic, Neoproterozoic, Phanerozoic). This normalization accounts for secular changes in the composition of Earth’s continental crust that reflect the emergence, growth, and subsequent differentiation of continental crust from the Archaean through the Phanerozoic. The evolution of continental crust influences the relative availability of trace and major elements weathered from land to the oceans, and thus the normalization removes this temporal variability, facilitating a comparison of Ni/Ti ratios in shales through time. Mean values for each time bin were bootstrap resampled (*n* = 10,000; Fig. 5) in Matlab® 2019b using the bootstrap function.

### Reporting summary

Further information on research design is available in the Nature Research Reporting Summary linked to this article.

## Supplementary information

Supplementary Information

Reporting Summary

Description of Additional Supplementary Files

Supplementary Data 1

Supplementary Data 2

Supplementary Data 3

Supplementary Data 4

## Data Availability

The sequence data analyzed in this study and accession numbers of genomes from the NCBI RefSeq database (https://www.ncbi.nlm.nih.gov/refseq/) are available in the open science framework repository, https://osf.io/yj7qb/?view_only=cc861929817e4913a7795cde10ae64fc.
