# Supplementary material for: Timing the evolution of antioxidant enzymes in cyanobacteria
Source: Nat Commun. 2021 Aug 6;12:4742. doi: 10.1038/s41467-021-24396-y (PMC8346466; doi:10.1038/s41467-021-24396-y)
Supplement: Supplementary file 1 — Supplementary Information [file 41467_2021_24396_MOESM1_ESM.pdf]

Timing the Evolution of Antioxidant Enzymes in Cyanobacteria

SUPPLEMENTARY INFORMATION

Joanne S. Boden[1*], Kurt O. Konhauser[2], Leslie J. Robbins[3,4], Patricia Sánchez-Baracaldo[1*]

[1]School of Geographical Sciences, University of Bristol, Bristol, BS8 1SS, United Kingdom

[2]Department of Earth and Atmospheric Sciences, University of Alberta, Edmonton, AB T6G 2E3, Canada

[3]Department of Earth and Planetary Sciences, Yale University, New Haven, CT, USA

[4]Department of Geology, University of Regina, Regina, SK S4S 0A2, Canada

*Correspondence:

Dr Patricia Sanchez-Baracaldo, School of Geographical Sciences, University of Bristol, Bristol, BS8 1SS, United Kingdom

Email: p.sanchez-baracaldo@bristol.ac.uk

Includes:

- Supplementary Discussion
- Supplementary Figures 1 to 12
- Supplementary Tables 1 to 4
- Supplementary Information References

SUPPLEMENTARY DISCUSSION

Horizontal Gene Transfer of *sodC* Among Cyanobacteria

Our phylogenetic analyses suggest (Fig. 1) that *sodC* has been laterally transferred between phyla on multiple occasions, so it would seem logical to assume the same could occur between different cyanobacteria. However, this does not seem to be the case for freshwater and terrestrial lineages because the majority of incongruence between our CuZnSOD phylogeny (Supplementary Fig. 5) and phylogenomic tree are indicative of HGT between marine strains. For example, the CuZnSODs of marine *Lyngbya majuscula* 3L and *Synechococcus* spp. are more closely related than expected based on the genome tree (Fig. 3; Supplementary Fig. 5). There is less known evidence of lateral gene transfer between cyanobacteria from terrestrial and freshwater habitats. For example, the evolutionary history of CuZnSODs from Nostocales, *Chroococcidiopsis* spp. and *Planktothrix* spp. broadly reflect patterns of vertical evolution with the species (Fig. 3; Supplementary Fig. 5).

Scenarios for an Earlier Emergence of NiSOD and Fe- and Mn-utilising SODs in Cyanobacteria

To find evidence of NiSOD, FeSOD or MnSOD before the GOE, our evolutionary trees would have to be interpreted in a slightly abstract way. For example, the *sodN* gene encoding NiSOD could have been present more than 800 mya if the gene from *Synechococcus* sp. PCC 7336 was horizontally transferred to microcyanobacteria and then horizontally transferred again from individual LPP to several different lineages of macrocyanobacteria (Supplementary Fig. 7). This is possible, given the commonality of *sodN* in marine lineages and the possibility of strains from similar niches undergoing HGT, but requires multiple lineages to be living in close proximity, which we cannot test with the current data. Similarly, Fe- and Mn-utilizing SODs could have been present ~2200 mya in the MRCA of *Thermosynechococcus elongatus* BP1 and Nostocales if

sodA or *sodB* were lost in seven independent lineages and retained only by two sister Macrocyanobacteria and Nostocales (Supplementary Fig. 9a). This seems unlikely given how frequently Mn- and Fe-utilizing SODs are found in modern cyanobacteria (Fig. 3). Therefore, we have included them in Fig. 4 but in paler colours to reflect their requirement for HGT.

SUPPLEMENTARY FIGURES

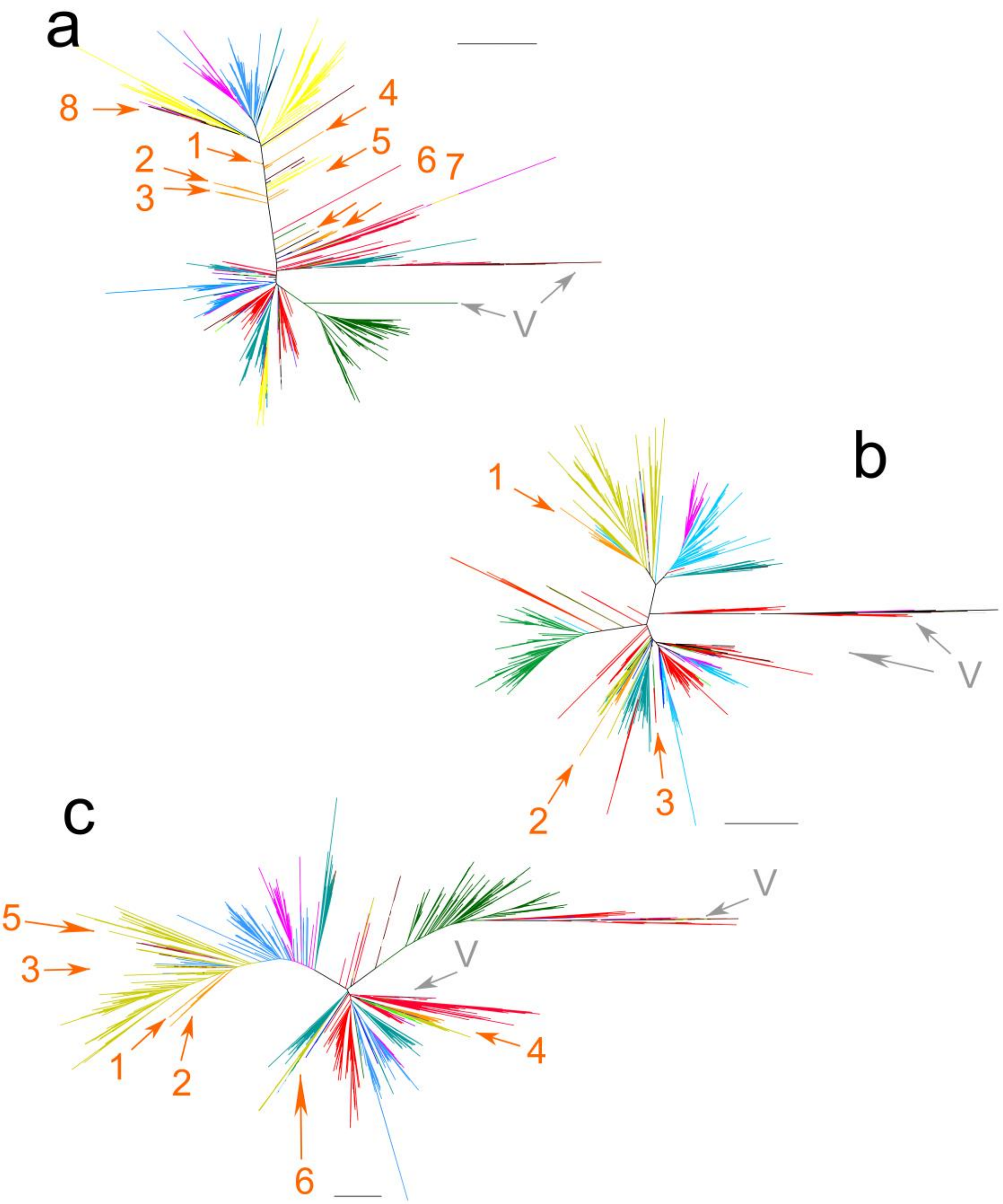

Supplementary Figure 1: Maximum likelihood phylogenies of bacterial FeSODs, MnSODs and cambialistic SODs generated from the same alignment of 224 amino acid positions using IQ-

TREE v1.6.1 [1], but with different random seeds and substitution models: a 839,059 with WAG+R10, b 874,629 with LG+R10 and c 36,982 with WAG+R10. WAG and LG refer to amino acid replacement matrices [2,3], whereas R10 refers to 10 categories of the FreeRate model [4] of estimating substitution rate heterogeneity across sites. Each substitution model was chosen independently by ModelFinder [5]. Scale bars represent the branch length required for 1 amino acid substitution per site in the phylogeny next to the bar. Arrows point out proteins from cyanobacteria (orange) and vampirovibionia (grey).

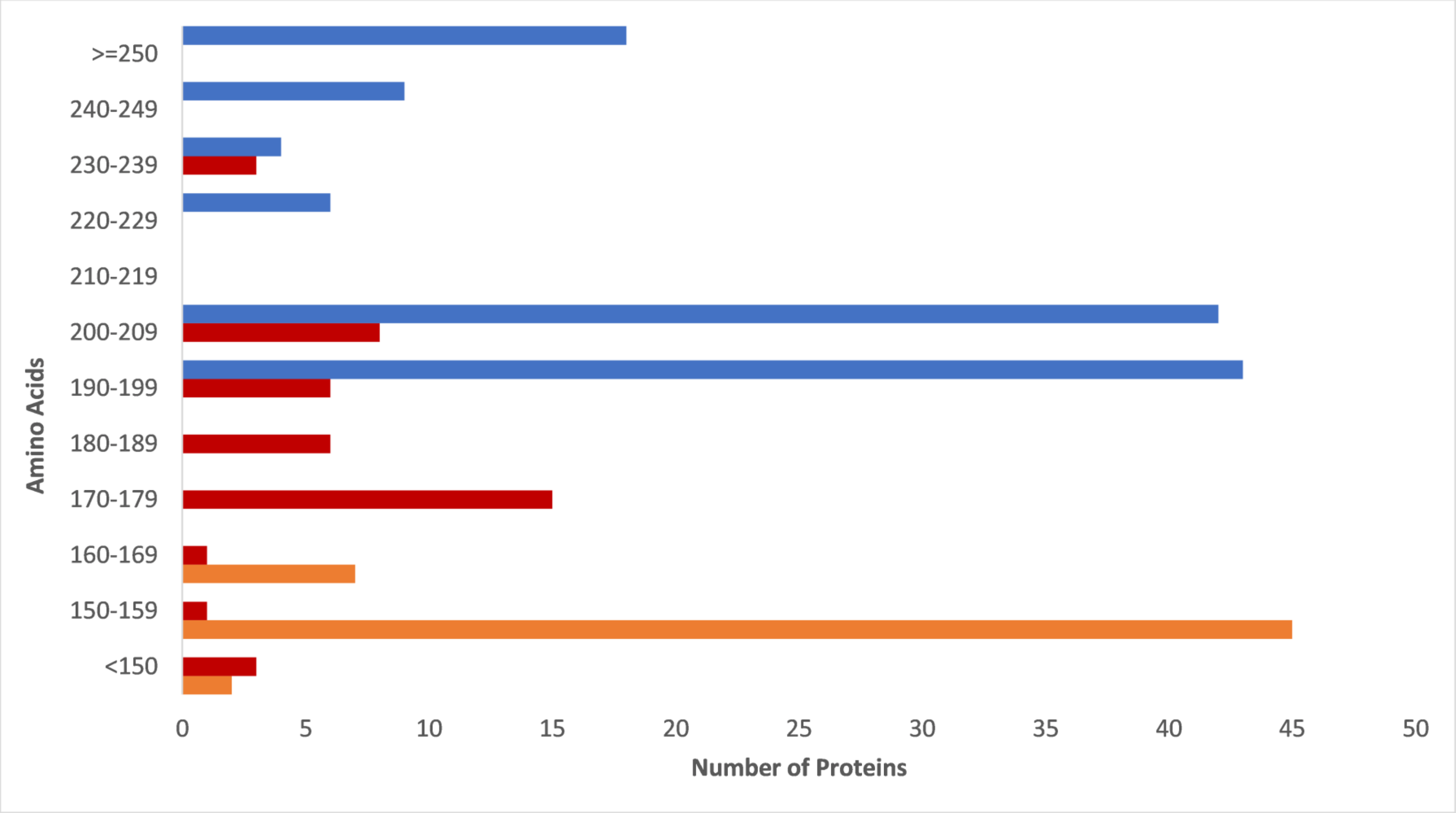

Supplementary Figure 2: Size of NiSOD (orange), CuZnSOD (red) and Fe- and Mn-utilising SODs (blue) in Cyanobacteria.

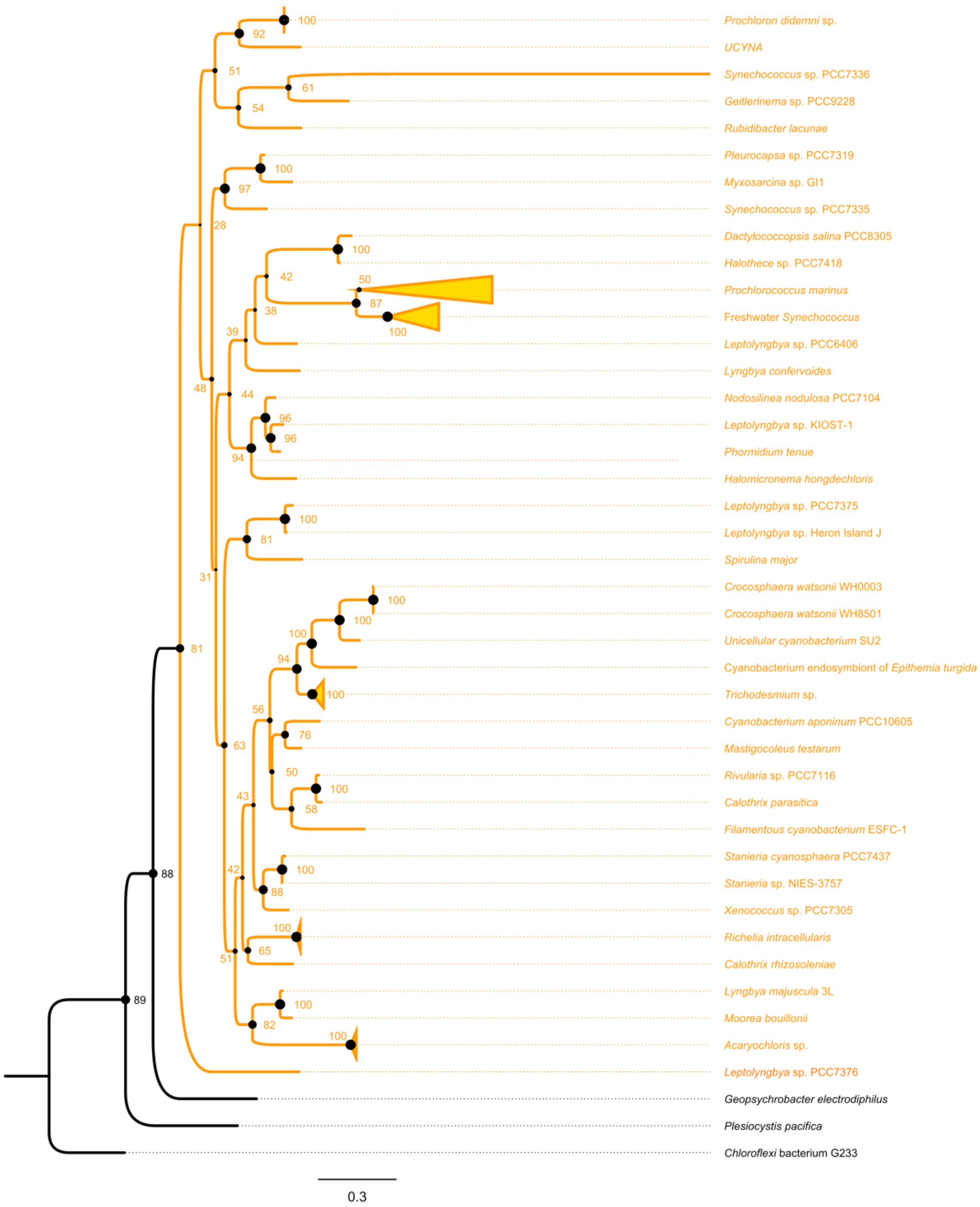

Supplementary Figure 3: Snapshot of Fig. 1a representing horizontal gene transfer of *sodN* between cyanobacteria and benthic marine deltaproteobacteria. The tree includes all

cyanobacterial sequences labelled with arrow 1 in Fig. 1. Taxon labels are coloured to represent cyanobacteria (orange) and other bacterial phyla (black). Branch labels describe UFBoot values. The higher the node value, the larger the node circle. The scale bar represents an average of 0.3 amino acid substitutions per site. Taxon labels are colored to represent cyanobacteria (orange) and other bacterial phyla (black). Branch labels describe UFBoot values. The higher the value, the larger the node circle. For methodology, see Fig. 1.

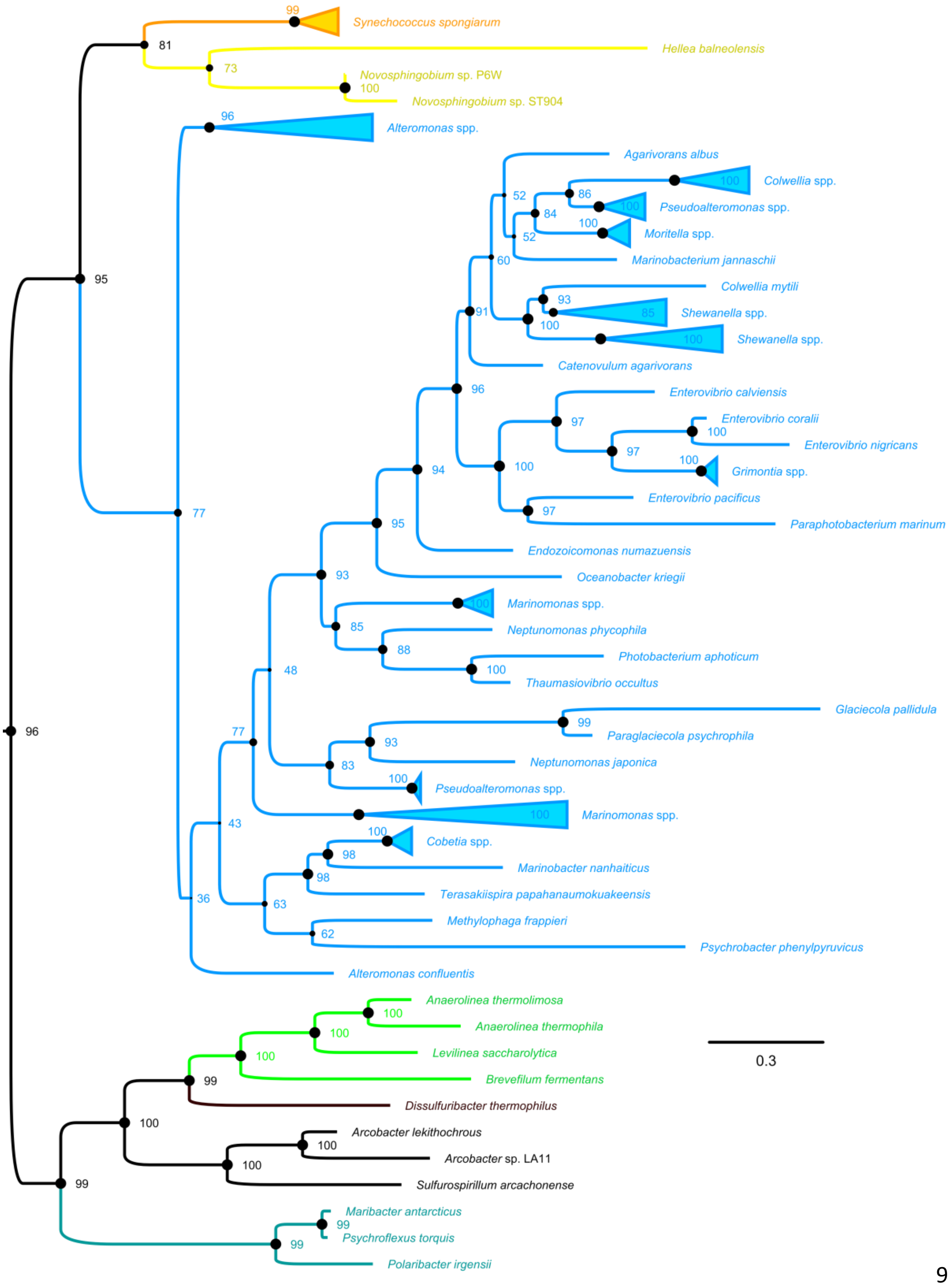

Synechococcus spongiarum
Hellea balneolensis
Novosphingobium sp. P6W
Novosphingobium sp. ST904
Alteromonas spp.
Agarivorans albus
Colwellia spp.
Pseudoalteromonas spp.
Moritella spp.
Marinobacterium jannaschii
Colwellia mytili
Shewanella spp.
Shewanella spp.
Catenovulum agarivorans
Enterovibrio calviensis
Enterovibrio coralii
Enterovibrio nigricans
Grimontia spp.
Enterovibrio pacificus
Paraphotobacterium marinum
Endozoicomonas numazuensis
Oceanobacter kriegii
Marinomonas spp.
Neptunomonas phycophila
Photobacterium aphoticum
Thaumasiovibrio occultus
Glaciecola pallidula
Paraglaciecola psychrophila
Neptunomonas japonica
Pseudoalteromonas spp.
Marinomonas spp.
Cobetia spp.
Marinobacter nanhaiticus
Terasakiispira papahanaumokuakeensis
Methylophaga frappieri
Psychrobacter phenylpyruvicus
Alteromonas confluentis
Anaerolinea thermolimosa
Anaerolinea thermophila
Levilinea saccharolytica
Brevefilum fermentans
Dissulfuribacter thermophilus
Arcobacter lekithochrous
Arcobacter sp. LA11
Sulfurospirillum arcachonense
Maribacter antarcticus
Psychroflexus torquis
Polaribacter irgensii
0.3

Supplementary Figure 4: Snapshot from Fig. 1a representing horizontal gene transfer of *sodN* between cyanobacterial endosymbionts and Proteobacteria. All cyanobacterial sequences are labelled with arrow 2 on Fig. 1. Taxon labels are colored to represent cyanobacteria (orange), alphaproteobacteria (yellow), betaproteobacteria (blue), chloroflexi (green), Bacteroidetes (turquoise), deltaproteobacteria (brown) and other bacterial phyla (black). Node labels are UFBoot values. The larger the node circle, the higher the value. The scale bar represents an average of 0.3 amino acid substitutions per site. For further methodological details, refer to Fig. 1.

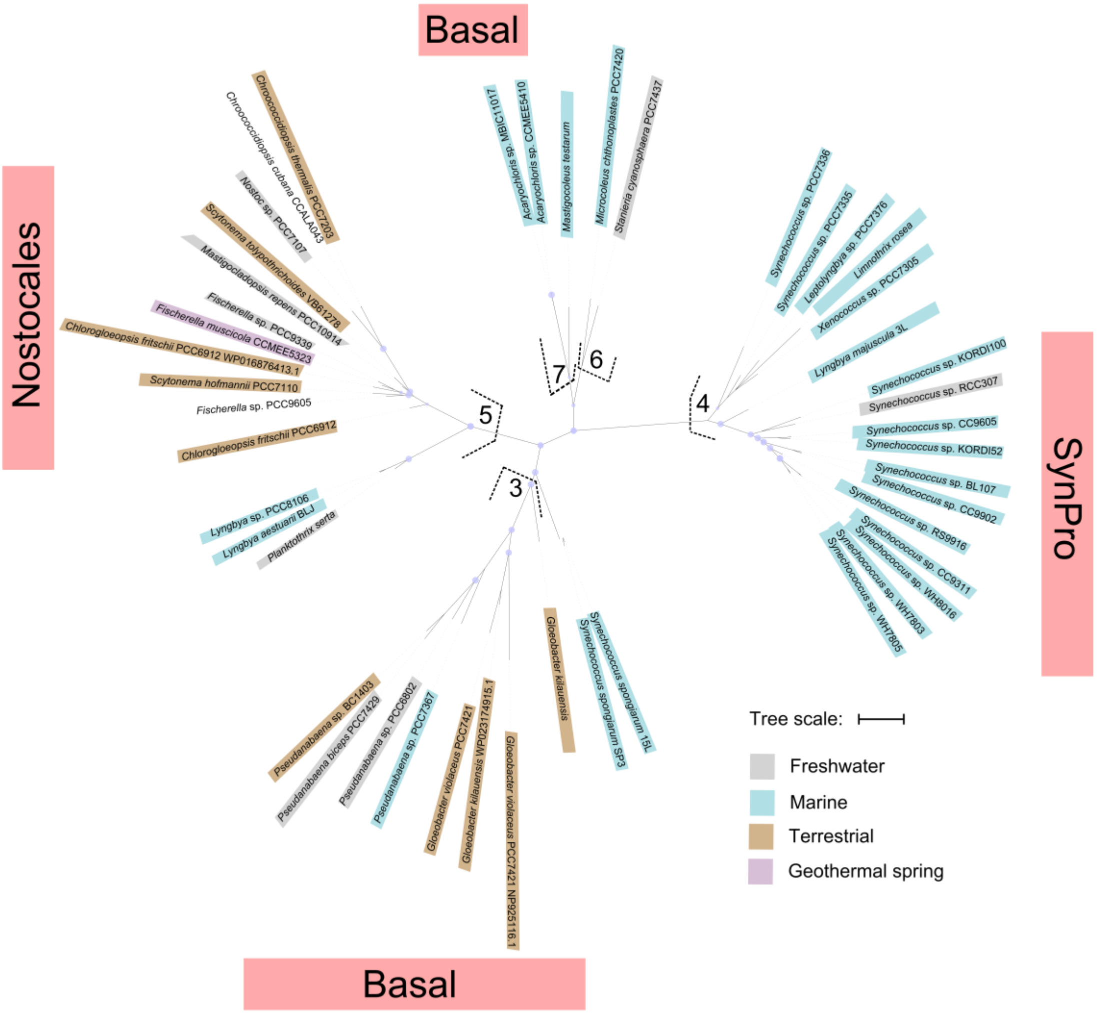

Supplementary Figure 5: Bayesian gene phylogeny of CuZnSOD in cyanobacteria. Alignment was generated in MAFFT (211 positions) and the phylogeny constructed using the GTR+CAT substitution model in Phylobayes 4.1 [6]. Node circles represent posterior probability values higher than 0.7 and numbered stars represent cyanobacterial sequences labelled with black arrows 3 to 7 in Fig. 1. Coloured backgrounds behind strain names represent the habitat they originated from, whereas the scale bar represents the branch length required for an average of 1 amino acid substitution per site.

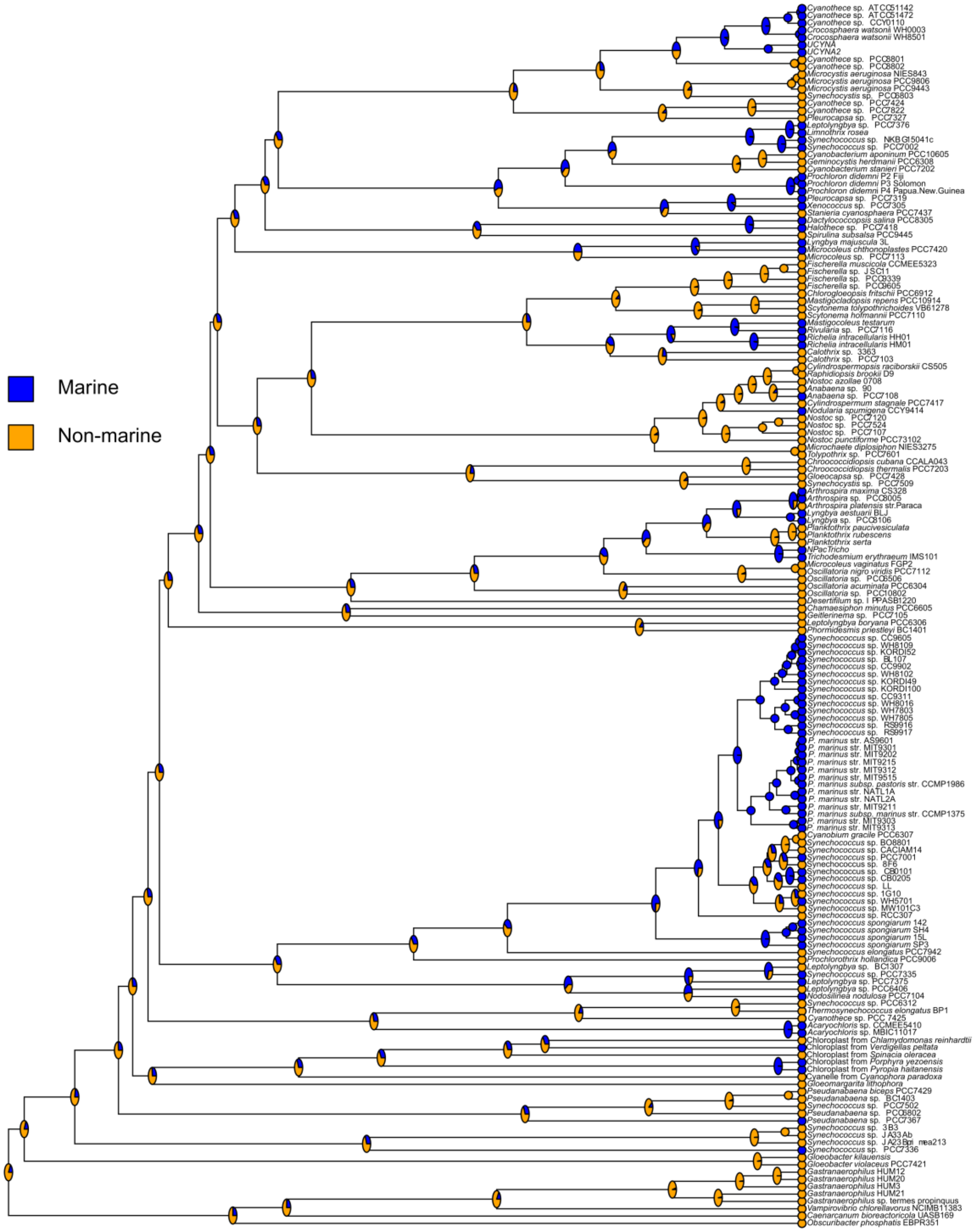
Marine
Non-marine
Cyanothece sp. ATCC51142
Cyanothece sp. ATCC51472
Cyanothece sp. CCY0110
Crocosphaera watsonii WH0003
Crocosphaera watsonii WH8501
UCYNA
UCYNA2
Cyanothece sp. PCC8801
Cyanothece sp. PCC8802
Microcystis aeruginosa NIES843
Microcystis aeruginosa PCC9806
Microcystis aeruginosa PCC9443
Synechocystis sp. PCC6803
Cyanothece sp. PCC7424
Cyanothece sp. PCC7822
Pleurocapsa sp. PCC7327
Leptolyngbya sp. PCC7376
Limnothrix rosea
Synechococcus sp. NKBG15041c
Synechococcus sp. PCC7002
Cyanobacterium aponinum PCC10605
Geminocystis herdmanii PCC6308
Cyanobacterium stanieri PCC7202
Prochloron didemni P2 Fiji
Prochloron didemni P3 Solomon
Prochloron didemni P4 Papua.New.Guinea
Pleurocapsa sp. PCC7319
Xenococcus sp. PCC7305
Stanieria cyanosphaera PCC7437
Dactylococcopsis salina PCC8305
Halothece sp. PCC7418
Spirulina subsalsa PCC9445
Lyngbya majuscula 3L
Microcoleus chthonoplastes PCC7420
Microcoleus sp. PCC7113
Fischerella muscicola CCMEE5323
Fischerella sp. JSC11
Fischerella sp. PCC9339
Fischerella sp. PCC9605
Chlorogloeopsis fritschii PCC6912
Mastigocladopsis repens PCC10914
Scytonema tolypothrichoides VB61278
Scytonema hofmannii PCC7110
Mastigocoleus testarum
Rivularia sp. PCC7116
Richelia intracellularis HH01
Richelia intracellularis HM01
Calothrix sp. 3363
Calothrix sp. PCC7103
Cylindrospermopsis raciborskii CS505
Raphidiopsis brookii D9
Nostoc azollae 0708
Anabaena sp. 90
Anabaena sp. PCC7108
Cylindrospermum stagnale PCC7417
Nodularia spumigena CCY9414
Nostoc sp. PCC7120
Nostoc sp. PCC7524
Nostoc sp. PCC7107
Nostoc punctiforme PCC73102
Microchaete diplosiphon NIES3275
Tolypothrix sp. PCC7601
Chroococcidiopsis cubana CCALA043
Chroococcidiopsis thermalis PCC7203
Gloeocapsa sp. PCC7428
Synechocystis sp. PCC7509
Arthrospira maxima CS328
Arthrospira sp. PCC8005
Arthrospira platensis str.Paraca
Lyngbya aestuarii BLJ
Lyngbya sp. PCC8106
Planktothrix paucivesiculata
Planktothrix rubescens
Planktothrix serta
NPacTricho
Trichodesmium erythraeum IMS101
Microcoleus vaginatus FGP2
Oscillatoria nigro viridis PCC7112
Oscillatoria sp. PCC6506
Oscillatoria acuminata PCC6304
Oscillatoria sp. PCC10802
Desertifilum sp. IPPASB1220
Chamaesiphon minutus PCC6605
Geitlerinema sp. PCC7105
Leptolyngbya boryana PCC6306
Phormidesmis priestleyi BC1401
Synechococcus sp. CC9605
Synechococcus sp. WH8109
Synechococcus sp. KORDI52
Synechococcus sp. BL107
Synechococcus sp. CC9902
Synechococcus sp. WH8102
Synechococcus sp. KORDI49
Synechococcus sp. KORDI100
Synechococcus sp. CC9311
Synechococcus sp. WH8016
Synechococcus sp. WH7803
Synechococcus sp. WH7805
Synechococcus sp. RS9916
Synechococcus sp. RS9917
P. marinus str. AS9601
P. marinus str. MIT9301
P. marinus str. MIT9202
P. marinus str. MIT9215
P. marinus str. MIT9312
P. marinus str. MIT9515
P. marinus subsp. pastoris str. CCMP1986
P. marinus str. NATL1A
P. marinus str. NATL2A
P. marinus str. MIT9211
P. marinus subsp. marinus str. CCMP1375
P. marinus str. MIT9303
P. marinus str. MIT9313
Cyanobium gracile PCC6307
Synechococcus sp. BO8801
Synechococcus sp. CACIAM14
Synechococcus sp. PCC7001
Synechococcus sp. 8F6
Synechococcus sp. CB0101
Synechococcus sp. CB0205
Synechococcus sp. LL
Synechococcus sp. 1G10
Synechococcus sp. WH5701
Synechococcus sp. MW101C3
Synechococcus sp. RCC307
Synechococcus spongiarum 142
Synechococcus spongiarum SH4
Synechococcus spongiarum 15L
Synechococcus spongiarum SP3
Synechococcus elongatus PCC7942
Prochlorothrix hollandica PCC9006
Leptolyngbya sp. BC1307
Synechococcus sp. PCC7335
Leptolyngbya sp. PCC7375
Leptolyngbya sp. PCC6406
Nodosilinea nodulosa PCC7104
Synechococcus sp. PCC6312
Thermosynechococcus elongatus BP1
Cyanothece sp. PCC 7425
Acaryochloris sp. CCMEE5410
Acaryochloris sp. MBIC11017
Chloroplast from Chlamydomonas reinhardtii
Chloroplast from Verdigellas peltata
Chloroplast from Spinacia oleracea
Chloroplast from Porphyra yezoensis
Chloroplast from Pyropia haitanensis
Cyanelle from Cyanophora paradoxa
Gloeomargarita lithophora
Pseudanabaena biceps PCC7429
Pseudanabaena sp. BC1403
Synechococcus sp. PCC7502
Pseudanabaena sp. PCC6802
Pseudanabaena sp. PCC7367
Synechococcus sp. 3B3
Synechococcus sp. JA33Ab
Synechococcus sp. JA23Bpi mea213
Synechococcus sp. PCC7336
Gloeobacter kilauensis
Gloeobacter violaceus PCC7421
Gastranaerophilus HUM12
Gastranaerophilus HUM20
Gastranaerophilus HUM3
Gastranaerophilus HUM21
Gastranaerophilus sp. termes propinquus
Vampirovibrio chlorellavorus NCIMB11383
Caenarcanum bioreactoricola UASB169
Obscuribacter phosphatis EBPR351

Supplementary Figure 6: Ancestral state reconstruction of habitat preference in cyanobacteria. Node labels are pie charts representing the probability of that node being marine (dark blue) or non-marine (yellow).

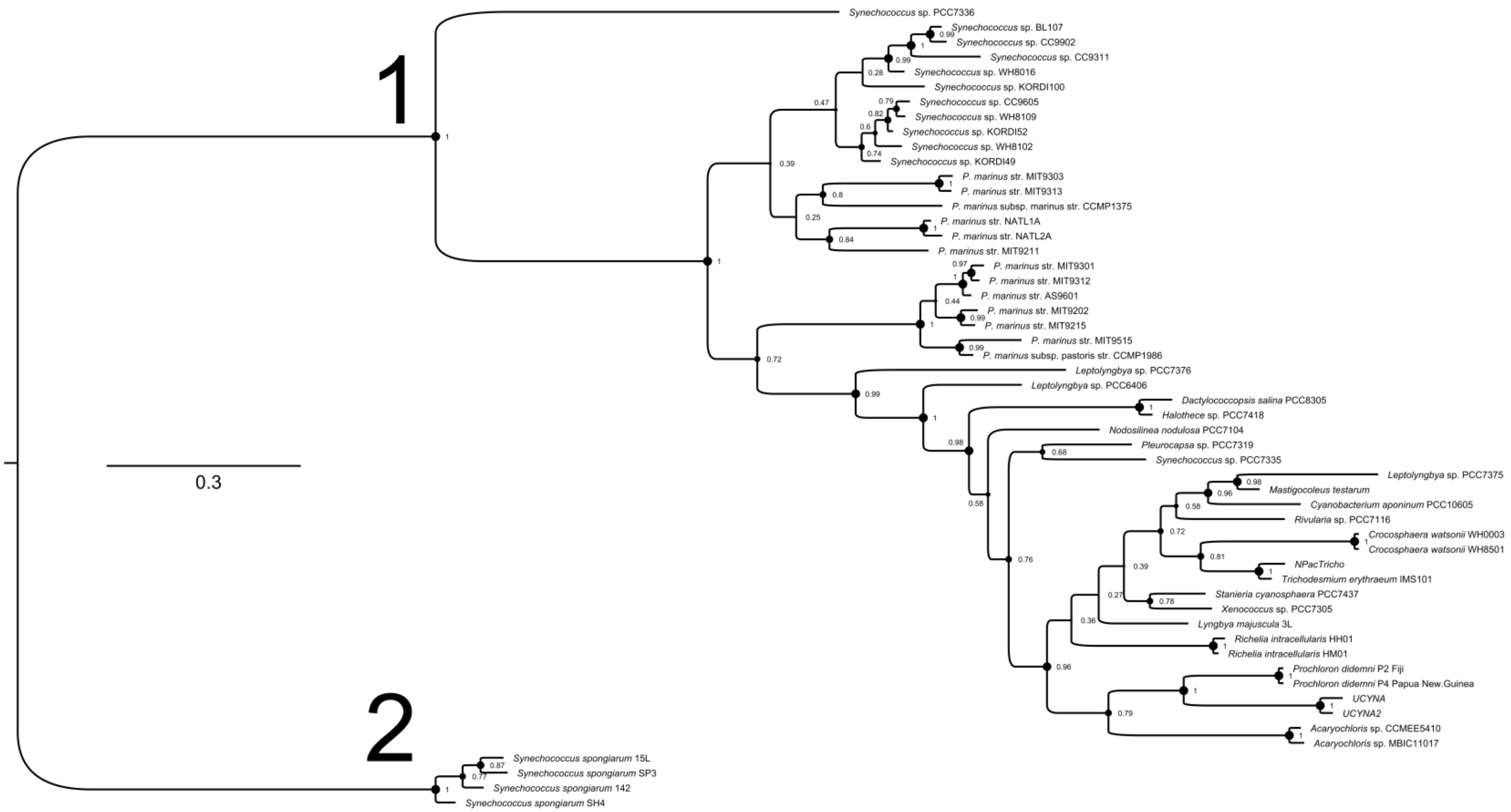

Supplementary Figure 7: Bayesian gene phylogeny of NiSOD in cyanobacteria. Alignment was generated in MAFFT (177 positions) and the phylogeny constructed using the LG+G4 substitution model in RevBayes v1.0.8 [7]. Node labels represent posterior probability. Higher values have

larger node circles and large numbers represent cyanobacteria labelled with black arrows 1 and 2 in Fig. 1. Branch lengths represent amino acid substitutions with the scale bar representing an average of 0.3 amino acid substitutions per site. The tree is rooted with cyanobacteria indicated by arrow number 2 in Fig. 1a.

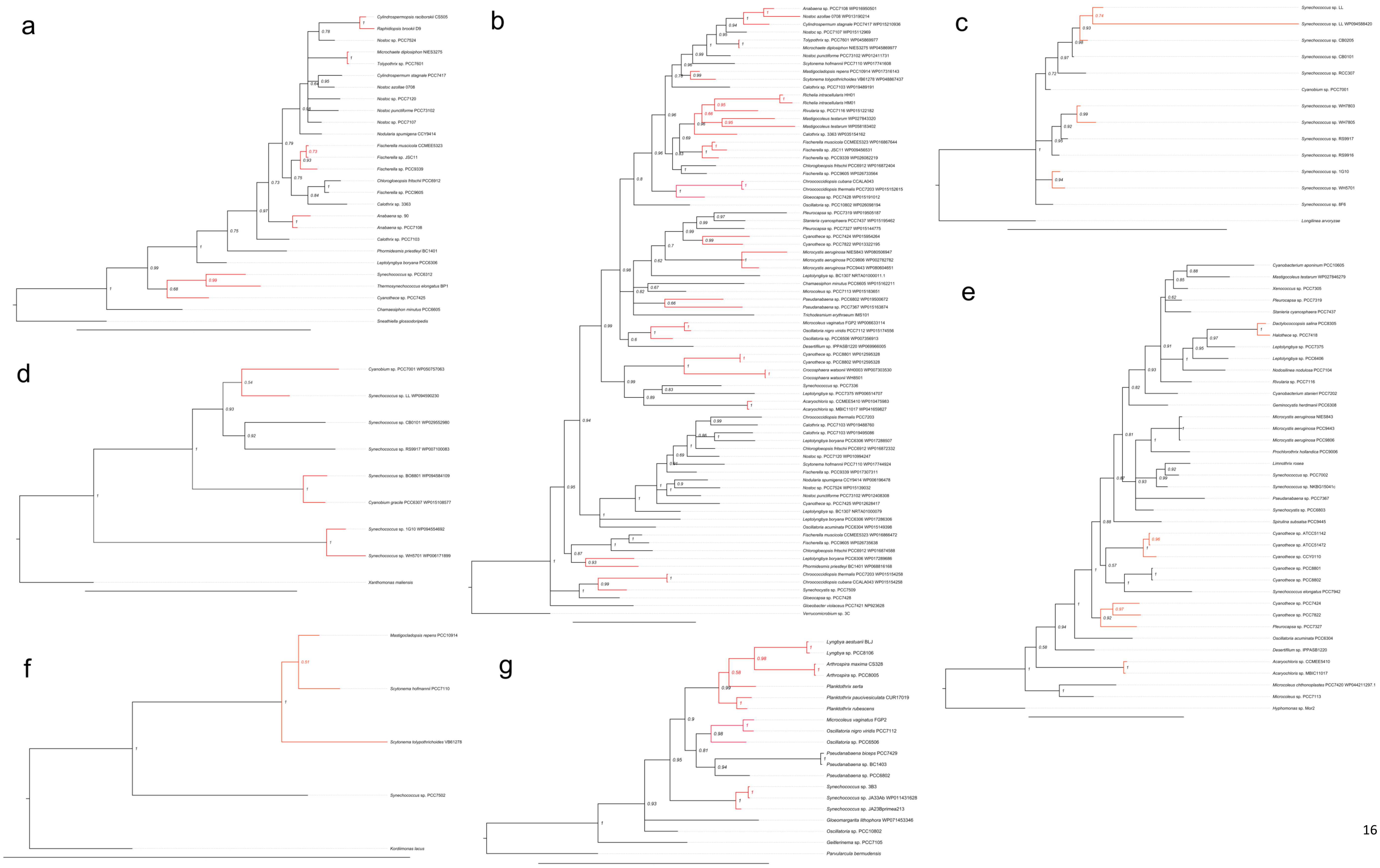
a
b
c
d
e
f
g

Supplementary Figure 8: Bayesian phylogenies of Mn Fe-utilising SODs. a homologs labelled with arrow 1 in Supplementary Figure 1c and some representatives labelled with arrow 2 in Supplementary Figure 1a and arrow 1 in Supplementary Figure 1b; b homologs labelled with arrow 7 in Supplementary Figure 1a as well as some but not all representatives labelled with arrow 2 in Supplementary Figure 1b and arrow 4 in Supplementary Figure 1c; c homologs labelled with arrow 4 in Supplementary Figure 1a, arrow 3 in Supplementary Figure 1c and some labelled with arrow 1 in Supplementary Figure 1b; d sequences labelled with arrow 6 in Supplementary Figure 1c as well as some representatives labelled 2 in Supplementary Figure 1b; e homologs labelled with arrow 3 in Supplementary Figure 1a and some of the sequences labelled with arrow 1 in Supplementary Figure 1b and arrow 2 in Supplementary Figure 1c; f homologs labelled with arrow 5 in Supplementary Figure 1a and some labelled with arrow 1 in Supplementary Figure 1b and arrow 2 in Supplementary Figure 1c; g homologs labelled with arrow 1 in Supplementary Figure 1c, arrow 1 in Supplementary Figure 1b and arrow 2 in Supplementary Figure 1a. Scale bars presented at the bottom of each panel represent an average of 0.5 amino acid substitutions per site. Node labels represent posterior probability values. All phylogenies were created in MrBayes v3.2.6 [8] using one of their predetermined fixed rate matrices with gamma-distributed rate variation across sites and a proportion of invariable sites. WAG was found to be the most appropriate matrix for all analyses. Clades of proteins with similar topology to strains in the genome tree are highlighted in red. Each tree was rooted using a closely related non-cyanobacterial outgroup identified from Supplementary Figure 1a.

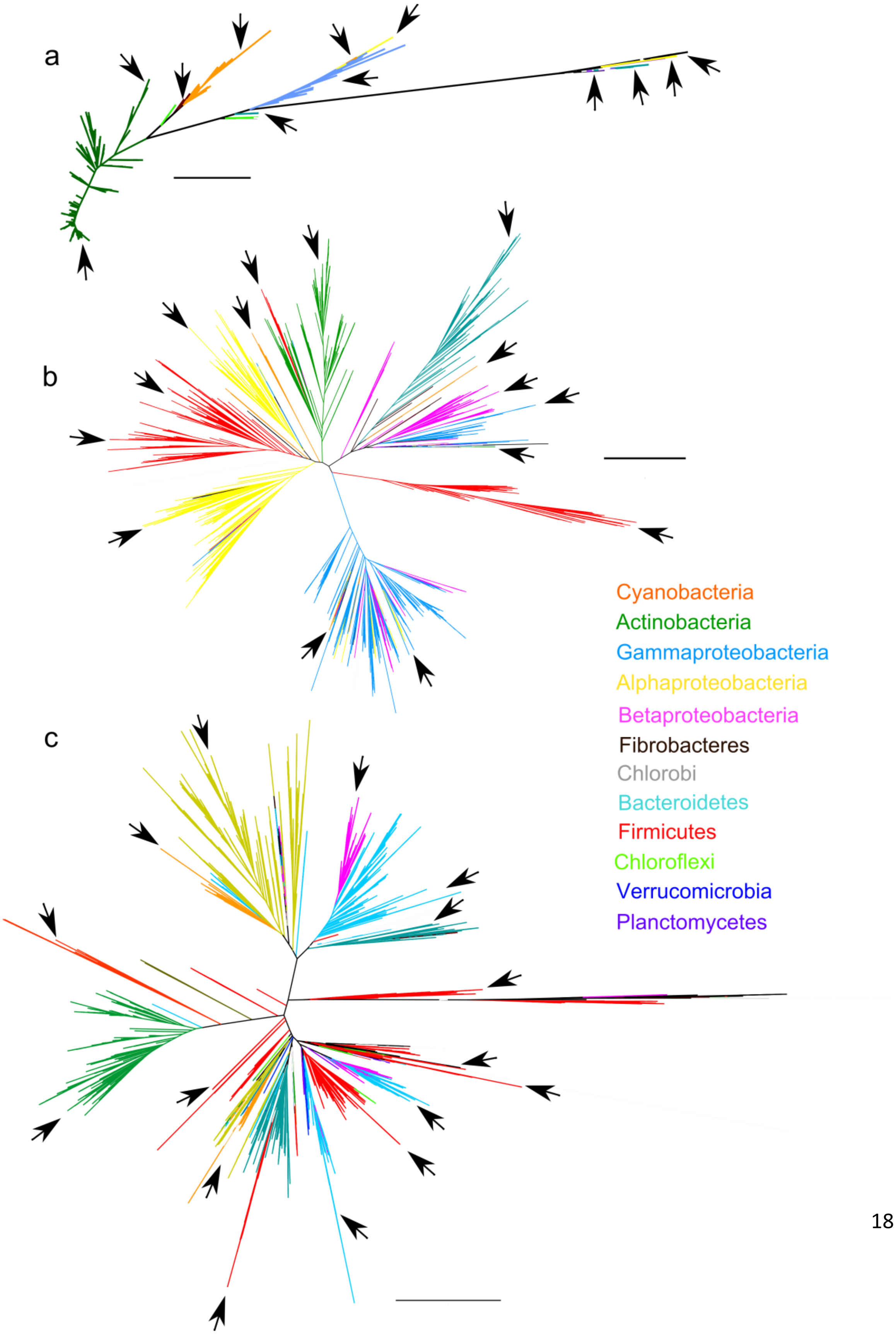
a
b
c
Cyanobacteria
Actinobacteria
Gammaproteobacteria
Alphaproteobacteria
Betaproteobacteria
Fibrobacteres
Chlorobi
Bacteroidetes
Firmicutes
Chloroflexi
Verrucomicrobia
Planctomycetes

Supplementary Figure 9: Phylogenetic positions of hits checked against the NCBI database in the bacterial phylogenies of a NiSOD, b CuZnSOD and c Fe and Mn-utilising SODs. Further details of hits including their name in the NCBI database are presented in Supplementary Data 2. Black arrows point to each hit that has been checked. Scale bars represent an average of 1 substitution per site.

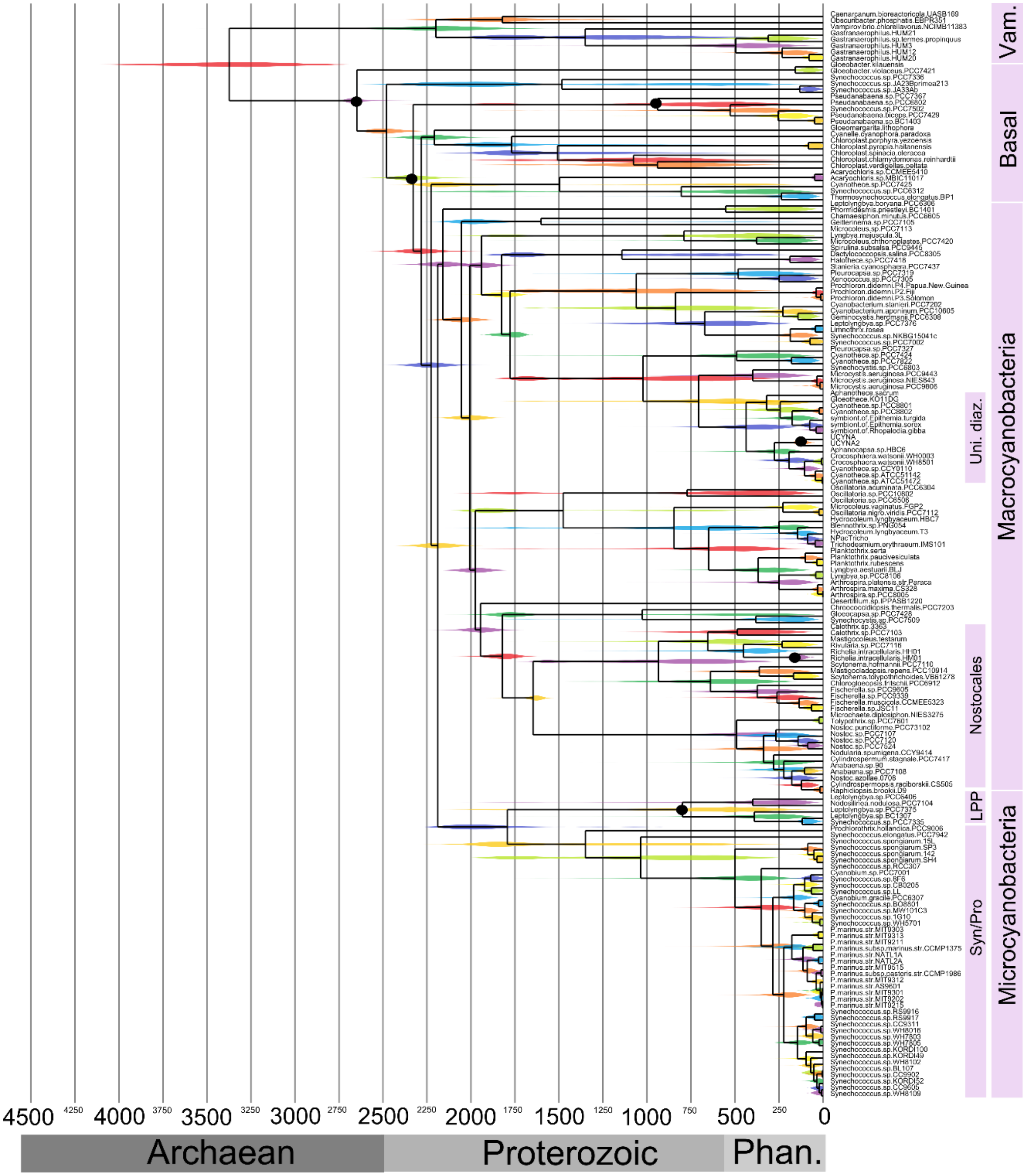

Vam.
Basal
Macrocyanobacteria
Uni. diaz.
Nostocales
LPP
Syn/Pro
Microcyanobacteria
4500
4250
4000
3750
3500
3250
3000
2750
2500
2250
2000
1750
1500
1250
1000
750
500
250
0
Archaean
Proterozoic
Phan.

Supplementary Figure 10: Variation in divergence times predicted by the Bayesian molecular clock implemented with uncorrelated gamma multipliers and 6 soft calibration points including first divergence of cyanobacteria between 2.32 Ga and 2.7 Ga.

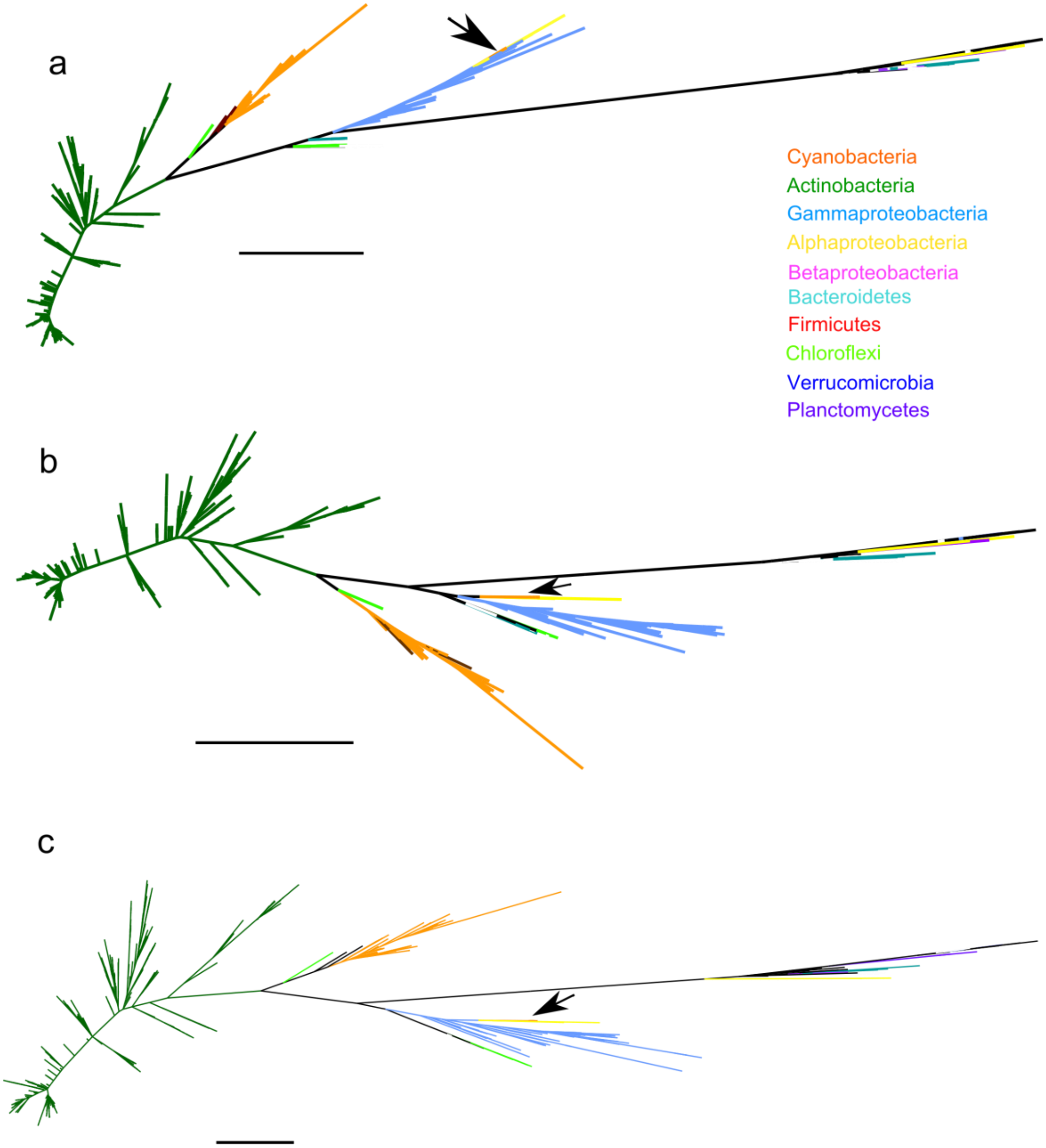

Supplementary Figure 11: Maximum Likelihood Phylogenies of Bacterial NiSOD Proteins constructed from the same alignment of 284 amino acid positions in IQ-TREE v1.6.1 [1] but with different random seeds and substitution models chosen by ModelFinder [5]: a 230,170 with LG+R8,

b 671,893 with LG+R8 and c 485,952 with LG+R7. LG refers to a general amino acid replacement matrix [3] and R refers to the FreeRate model of heterogeneity across sites in the alignment [4] with 7 or 8 categories. Scale bars represent an average of 1 amino acid substitution per site. Arrows point out *Synechococcus spongiarum* of the phylum Cyanobacteria.

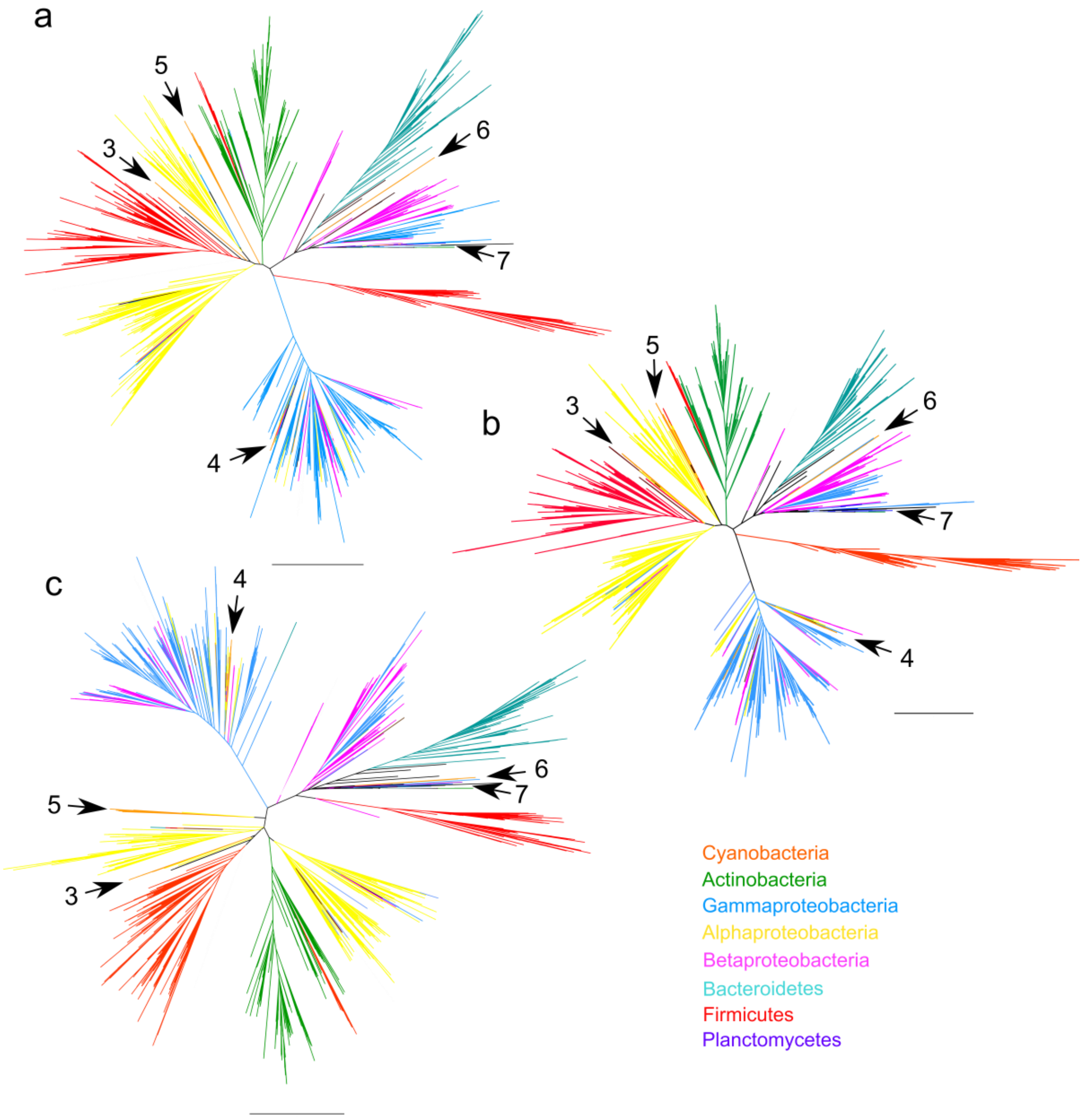

Supplementary Figure 12: Maximum likelihood phylogenies of bacterial CuZnSOD proteins constructed from the same alignment of 208 amino acid positions using IQ-TREE v1.6.1 [1], but with different random seeds of a 737,859, b 487,350 and c 138,936. Substitution models were chosen independently by ModelFinder [5], but all phylogenies utilized WAG [2] with 10 categories of

the FreeRate model [4] to estimate heterogeneity across sites. Scale bars represent the branch length required for on average 1 amino acid substitution per site in the phylogeny immediately above the bar. Arrows point out cyanobacterial proteins from *Gloeobacter* sp. and *Pseudanabaena* sp. (3), *Synechococcus* sp. (4), Nostocales, *Planktothrix* sp. and *Lyngbya* sp. (5), *Microcoleus chthonoplastes* (6) and *Acaryochloris* sp. (7).

SUPPLEMENTARY TABLES

Supplementary Table 1: Completeness of genomes with the *sodN* gene encoding NiSOD, but not the *sodX* gene encoding NiSOD's maturation protease. Completeness was measured with BUSCO v3.0.3 [9] using cyanobacteria lineage data. Genus and species names are italicized.

Cyanobacterium	Lifestyle	Genome Completeness
Leptolyngbya sp. PCC 7375	Free-living	98 %
Leptolyngbya sp. BC1307	Free-living	98%
Synechococcus sp. *PCC 7336*	Free-living	92 %
P. subspecies pastoris strain CCMP1986	Free-living	93 %
Prochlorococcus marinus strain MIT9515	Free-living	93 %
Leptolyngbya sp. PCC 7376	Free-living	96%
Synechococcus spongiarum 142	Symbiont	72 %
Synechococcus spongiarum SP3	Symbiont	85 %
Synechococcus spongiarum 15L	Symbiont	75 %
Synechococcus spongiarum SH4	Symbiont	60%

Supplementary Table 2: Results of tBLASTN searches for Fe- and Mn-utilising SODs in Vampirovibrionia. Genus and species names are italicised.

Strain	Percent Identity	E Value	Alignment Length	Nucleotide ID	Sequence Start	Sequence End
Obscuribacter phosphatis EBPR351	26	3.55E-09	211	MOYA01000002.1	565837	565220
Obscuribacter phosphatis EBPR351	26	5.22E-07	188	MOYA01000001.1	1603143	1602610
Vampirovibrio chlorellavorus NCIMB11383	31	2.40E-12	192	LAPX01000004.1	553878	554426
Vampirovibrio chlorellavorus NCIMB11383	25	1.51E-06	185	LAPX01000007.1	270177	270692
Caenarcanum bioreactoricola UASB169	32	8.16E-29	185	MOYB01000001.1	109221	108673

Supplementary Table 3: Query sequences used to find SOD isoforms in bacterial genomes. Where multiple query sequences are reported for the same isoform, one of the 5 query sequences were used for either BLASTP or tBLASTN (depending on the genome information available for that taxon). If hit(s) were found with e values less than $1x10^{-5}$, no other query sequences were used. If not, another query sequence was chosen and the relevant BLAST search repeated. If appropriate hits were still not identified, this process was repeated up to 5 times (after which the gene was assumed not to be present in the genome). A single query sequence was utilized for CuZnSOD to prevent proteins with other annotated functions being found. Genus and species names are italicized.

SOD Isoform	Strain	NCBI ID [web location]
NiSOD	*Prochloroccus marinus* str. MIT9202	WP_033829816.1 [https://www.ncbi.nlm.nih.gov/protein/WP_033829816.1/]
	Synechococcus sp. CC9311	WP_011618696.1 [https://www.ncbi.nlm.nih.gov/protein/WP_011618696.1]
	Synechococcus spongiarum SP3	WP_046840990.1 [https://www.ncbi.nlm.nih.gov/protein/WP_046840990.1?report=genpept]
	Prochlorococcus marinus subsp. marinus str. CCMP1375	NP_87579.1 [https://www.ncbi.nlm.nih.gov/protein/NP_875759.1?report=genpept]
	Crocosphaera watsonii WH8501	WP_007306654.1 [https://www.ncbi.nlm.nih.gov/protein/494517196]
CuZnSOD	*Gloeobacter kilauensis*	WP_023172368.1 [https://www.ncbi.nlm.nih.gov/protein/WP_023172368.1]
Mn- and Fe-utilising SODs	*Trichodesmium erythreum* IMS101	WP_011613835.1 [https://www.ncbi.nlm.nih.gov/protein/WP_011613835.1]
	Nostoc sp. PCC 7120	AAD51417.1 [https://www.ncbi.nlm.nih.gov/protein/AAD51417.1]
	Nostoc sp. PCC 7107	WP_015112374.1 [https://www.ncbi.nlm.nih.gov/protein/WP_015112374.1]
	Nostoc sp. PCC 7107	WP_015112969.1 [https://www.ncbi.nlm.nih.gov/protein/WP_015112969.1]
	Synechococcus sp. BO 8801	WP_094585426.1 [https://www.ncbi.nlm.nih.gov/protein/WP_094585426.1]

Supplementary Table 4: Isolation location of cyanobacteria used in this study.

Habitat	Number of Strains
Marine	73
Hypersaline lake	1
Total:	74
Freshwater	57
Terrestrial	13
Brackish	1
Geothermal springs	6
Total:	77
Miscellaneous	2

SUPPLEMENTARY INFORMATION REFERENCES

1 Trifinopoulos, J., Nguyen, L. T., von Haeseler, A. & Minh, B. Q. W-IQ-TREE: A fast online phylogenetic tool for maximum likelihood analysis. *Nucleic Acids Res.* **44**, W232-235 (2016).

2 Whelan, S. & Goldman, N. A general empirical model of protein evolution derived from multiple protein families using a maximum-likelihood approach. *Mol. Biol. Evol.* **18**, 691-699 (2001).

3 Le, S. Q. & Gascuel, O. An improved general amino acid replacement matrix. *Mol. Biol. Evol.* **25**, 1307-1320 (2008).

4 Yang, Z. A space-time process model for the evolution of DNA sequences. *Genetics* **139**, 993-1005 (1995).

5 Kalyaanamoorthy, S., Minh, B. Q., Wong, T. K. F., von Haeseler, A. & Jermiin, L. S. ModelFinder: Fast model selection for accurate phylogenetic estimates. *Nat. Methods* **14**, 587-591 (2017).

6 Lartillot, N., Lepage, T. & Blanquart, S. PhyloBayes 3: a Bayesian software package for phylogenetic reconstruction and molecular dating. *Bioinformatics* **25**, 2286-2288 (2009).

7 Hohna, S. *et al.* RevBayes: Bayesian phylogenetic inference using graphical models and an interactive model-specification language. *Syst. Biol.* **65**, 726-736 (2016).

8 Ronquist, F. *et al.* MrBayes 3.2: Efficient Bayesian phylogenetic inference and model choice across a large model space. *Syst. Biol.* **61**, 539-542 (2012).

9 Simao, F. A., Waterhouse, R. M., Ioannidis, P., Kriventseva, E. V. & Zdobnov, E. M. BUSCO: Assessing genome assembly and annotation completeness with single-copy orthologs. *Bioinformatics* **31**, 3210-3212 (2015).
