# Supplementary material for: Timing the evolution of antioxidant enzymes in cyanobacteria
Source: Nat Commun. 2021 Aug 6;12:4742. doi: 10.1038/s41467-021-24396-y (PMC8346466; doi:10.1038/s41467-021-24396-y)
Supplement: Supplementary file 2 — Reporting Summary [file 41467_2021_24396_MOESM2_ESM.pdf]

nature research

Corresponding author(s): Patricia Sanchez-Baracaldo

Last updated by author(s): Jun 6, 2021

Reporting Summary

Nature Research wishes to improve the reproducibility of the work that we publish. This form provides structure for consistency and transparency in reporting. For further information on Nature Research policies, see our Editorial Policies and the Editorial Policy Checklist.

Statistics

For all statistical analyses, confirm that the following items are present in the figure legend, table legend, main text, or Methods section.

n/a	Confirmed	
☒	☐	The exact sample size (*n*) for each experimental group/condition, given as a discrete number and unit of measurement
☒	☐	A statement on whether measurements were taken from distinct samples or whether the same sample was measured repeatedly
☒	☐	The statistical test(s) used AND whether they are one- or two-sided<br>*Only common tests should be described solely by name; describe more complex techniques in the Methods section.*
☒	☐	A description of all covariates tested
☐	☒	A description of any assumptions or corrections, such as tests of normality and adjustment for multiple comparisons
☐	☒	A full description of the statistical parameters including central tendency (e.g. means) or other basic estimates (e.g. regression coefficient) AND variation (e.g. standard deviation) or associated estimates of uncertainty (e.g. confidence intervals)
☒	☐	For null hypothesis testing, the test statistic (e.g. *F*, *t*, *r*) with confidence intervals, effect sizes, degrees of freedom and *P* value noted<br>*Give P values as exact values whenever suitable.*
☐	☒	For Bayesian analysis, information on the choice of priors and Markov chain Monte Carlo settings
☒	☐	For hierarchical and complex designs, identification of the appropriate level for tests and full reporting of outcomes
☒	☐	Estimates of effect sizes (e.g. Cohen's *d*, Pearson's *r*), indicating how they were calculated

Our web collection on statistics for biologists contains articles on many of the points above.

Software and code

Policy information about availability of computer code

Data collection	Genome sequences and query sequences for BLASTP analyses were collected manually from the NCBI RefSeq database (https://www.ncbi.nlm.nih.gov/refseq/).
Data analysis	Data were analysed with the following software:<br>BLAST v2.10.0+<br>MAFFT v7.427<br>IQTREE v1.6.1<br>Phylobayes v4.1<br>RevBayes v1.0.8<br>R v3.4.4<br>Matlab 2019a<br>Matlab 2019b

For manuscripts utilizing custom algorithms or software that are central to the research but not yet described in published literature, software must be made available to editors and reviewers. We strongly encourage code deposition in a community repository (e.g. GitHub). See the Nature Research guidelines for submitting code & software for further information.

Data

Policy information about availability of data

All manuscripts must include a data availability statement. This statement should provide the following information, where applicable:

- Accession codes, unique identifiers, or web links for publicly available datasets
- A list of figures that have associated raw data
- A description of any restrictions on data availability

The sequence data analysed in this study and accession numbers of genomes from the NCBI RefSeq database (https://www.ncbi.nlm.nih.gov/refseq/) are available in the open science framework repository, https://osf.io/yj7qb/?view_only=cc861929817e4913a7795cde10ae64fc.

Field-specific reporting

Please select the one below that is the best fit for your research. If you are not sure, read the appropriate sections before making your selection.

☒ Life sciences ☐ Behavioural & social sciences ☐ Ecological, evolutionary & environmental sciences

For a reference copy of the document with all sections, see nature.com/documents/nr-reporting-summary-flat.pdf

Life sciences study design

All studies must disclose on these points even when the disclosure is negative.

Sample size	In this study, we select a sample of superoxide dismutase enzymes with known amino acid sequences and use them to search for homologs in a sample of bacterial genomes. The size of neither sample was established with statistical methods. Instead, the sample of superoxide dismutase enzymes was chosen based on experimental evidence confirming their function, as described in the Methods section of the manuscript. By selecting as many proteins as possible with experimentally-verified functions, we reduce the chance of finding homologs with different biological roles.<br><br>Our sample of bacterial genomes were selected to maximise the representation of bacterial diversity. Therefore, we downloaded all that were available on the NCBI RefSeq database (http://www.ncbi.nlm.nih.gov/refseq/) in 2018. Although other genomes could have been sourced from other databases, we believe this to be sufficient because it includes representatives from most major bacterial phyla.<br><br>The sample of cyanobacterial genomes used to construct the molecular clock presented in Figure 3 were chosen manually to ensure a broad coverage of the cyanobacterial phylum. To do this, we evaluated previously published cyanobacterial evolutionary trees (e.g. Sanchez-Baracaldo et al, 2015, Shih et al, 2013) and selected 164 strains which represented all major groups from marine, freshwater and terrestrial lineages. This sample size was small enough to enable the relevant analyses (e.g. bayesian molecular clocks and phylogenetic reconstruction) to be completed in a suitable time-frame without biasing out results.
Data exclusions	Some genomes of cyanobacteria and vampirovibrionia were not incorporated into the bayesian molecular clock to save computational time. The exclusion criteria were not pre-established, but we aimed to include a total of 125-175 genomes based on previous experience. Hundreds more cyanobacterial genomes were available at the time, but we chose to discount them because information at the time suggested that they were very close relatives of other species included in the analysis. Close relatives provide little information about events which happened hundreds of millions of years ago. As our study aimed to investigate the evolution of superoxide dismutase enzymes over a geological timescale, very close relatives were not required.
Replication	The evolutionary tree of cyanobacteria was replicated three times using the same scripts and data but different initial random number seeds. All resulting trees exhibited similar topology.<br><br>Evolutionary trees constructed from the CuZnSODs, NiSODs, FeSODs, MnSODs and cambialistic SODs found in 15,899 bacterial genomes were each replicated three times using the same alignments and commands. Overall topology of all three CuZnSOD phylogenies and all three NiSOD phylogenies were similar and are presented in the Supplementary information. Unfortunately, topology of the trees containing Fe- and Mn-utilising SODs were different. These differences are presented in Supplementary Fig. 1 and were accounted for in future analyses of FeSOD, MnSOD and cambialistic SOD evolution.<br><br>Two sets of Bayesian molecular clocks were run with different calibration points, but each were replicated four times. This resulted in four replicate analyses which were compared to estimate convergence. All replicates converged on a similar result.
Randomization	Randomisation is incorporated into the software used for phylogenetic reconstruction
Blinding	Investigators were not blinded during data analysis. The same author who constructed evolutionary trees of NiSOD, CuZnSOD and Mn- and Fe-utilising SODs also compared their topology to the species tree of cyanobacteria to estimate when they evolved. Each tree was easily recognisable, so it was not possible to blind the author to the SOD isoform they were investigating.

Reporting for specific materials, systems and methods

We require information from authors about some types of materials, experimental systems and methods used in many studies. Here, indicate whether each material, system or method listed is relevant to your study. If you are not sure if a list item applies to your research, read the appropriate section before selecting a response.

Materials & experimental systems

n/a	Involved in the study
☒	☐ Antibodies
☒	☐ Eukaryotic cell lines
☒	☐ Palaeontology and archaeology
☒	☐ Animals and other organisms
☒	☐ Human research participants
☒	☐ Clinical data
☒	☐ Dual use research of concern

Methods

n/a	Involved in the study
☒	☐ ChIP-seq
☒	☐ Flow cytometry
☒	☐ MRI-based neuroimaging
