# Supplementary material for: Timing the evolution of antioxidant enzymes in cyanobacteria
Source: Nat Commun. 2021 Aug 6;12:4742. doi: 10.1038/s41467-021-24396-y (PMC8346466; doi:10.1038/s41467-021-24396-y)
Supplement: Supplementary file 3 — Description of Additional Supplementary Files [file 41467_2021_24396_MOESM3_ESM.pdf]

Description of Additional Supplementary Files

File Name: Supplementary Data 1
Description: Results of BLAST Searches for SOD isoforms in cyanobacteria from different habitats. Where more than 1 hit is found, statistics are reported for the hit with the smallest E value. Iden. Identity. Strains from 'fresh', 'geothermal spring' and 'terrestrial' habitats were considered 'non-marine' for ancestral state reconstructions of habitat preference.

File Name: Supplementary Data 2
Description: Accession numbers of proteins available from the NCBI RefSeq database (https://www.ncbi.nlm.nih.gov/refseq/) and incorporated into Figure 1.

File Name: Supplementary Data 3
Description: Detailed information on the identity of a representative subsample of proteins found in BLASTP searches for NiSOD, CuZnSOD and Fe- and Mn-utilising SODs in 15,899 strains of bacteria. The hits for each SOD isoform are from distant parts of their respective phylogenies represented in Supplementary Fig. 9.

File Name: Supplementary Data 4
Description: Sources for the data presented in Fig. 5. Each record is presented alongside the sample name, source, geographical location, geological formation and the associated age and values for Ni and % TiO_2. Ga. billions of years ago.
